# A Latent Markov Model for Noninvariant Measurements: An Application to Interaction Log Data From Computer-Interactive Assessments

**DOI:** 10.1017/psy.2025.10029

**Published:** 2025-08-26

**Authors:** Hyeon-Ah Kang

**Affiliations:** Department of Educational Psychology, University of Texas at Austin, Austin, TX, USA

**Keywords:** computerized assessments, interaction log, latent Markov model (LMM), longitudinal measurement invariance, measurement noninvariance, process data, transition analysis

## Abstract

The latent Markov model (LMM) has been increasingly used to analyze log data from computer-interactive assessments. An important consideration in applying the LMM to assessment data is measurement effects of items. In educational and psychological assessment, items exhibit distinct psychometric qualities and induce systematic variance to assessment outcome data. The current development in LMM, however, assumes that items have uniform effects and do not contribute to the variance of measurement outcomes. In this study, we propose a refinement of LMM that relaxes the measurement invariance constraint and examine empirical performance of the new framework through numerical experimentation. We modify the LMM for noninvariant measurements and refine the inferential scheme to accommodate the event-specific measurement effects. Numerical experiments are conducted to validate the proposed inference methods and evaluate the performance of the new framework. Results suggest that the proposed inferential scheme performs adequately well in retrieving the model parameters and state profiles. The new LMM framework demonstrated reliable and stable performance in modeling latent processes while appropriately accounting for items’ measurement effects. Compared with the traditional scheme, the refined framework demonstrated greater relevance to real assessment data and yielded more robust inference results when the model was ill-specified. The findings from the empirical evaluations suggest that the new framework has potential for serving large-scale assessment data that exhibit distinct measurement effects.

## 1 Introduction

As computers are increasingly used in educational and psychological assessments, interaction log data (e.g., response times, mouse clickstreams, action counts) have become readily accessible and afforded numerous research opportunities. The interaction log data, also known as process data in educational assessment, record individual’s interaction with an operation system and have been used to inform respondents’ behavioral patterns and mental modes (Greiff et al., 2016; He et al., 2023; Kang et al., 2024; Welling et al., 2024). In education, the interaction data have been further used to predict students’ future performance (Qiu et al., 2018; Waheed et al., 2023), refine assessment designs and interventions (Dunbar et al., 2014; Kuo & Wu, 2013; Mislevy et al., 1999).

One way to model interaction log data is to apply the latent Markov model (LMM; Baum & Petrie, 1966; Wiggins, 1955, 1973). The model describes individual’s interaction outcomes as a cross-sectional time-series and posits a latent state sequence to explain the variance of manifest observations. The model can describe dynamics of temporal outcomes while offering flexibility in modeling various indicator variables. In the field of education, LMM has been used to model students’ learning behaviors (Chen et al., 2019; Geigle & Zhai, 2017; Shih et al., 2010; Tang et al., 2021), mental modes (Kang et al., 2024; Molenaar et al., 2016, 2019), problem-solving strategies (Tang, 2024; Xiao et al., 2021), and affective states (Fwa & Marshall, 2018; Maqsood et al., 2022).

An important consideration in applying the LMM to assessment data is invariance of measurement (Cappé et al., 2005, chapter 14)—the measurement process that generates manifest observations from latent states must remain constant across occasions. When construed for assessment data, the assumption of measurement invariance (MI) means that the measurement stimuli that prompt responses (e.g., items, problems, tasks, and questions) have homogeneous psychometric qualities and do not induce variance to the outcome data. The MI assumption ensures that longitudinal variance in the outcome data is explained by the evolution of underlying latent states. If the measurement system fluctuates over occasions — termed as measurement noninvariance (MNI)—, the difference in the outcome data cannot be fully attributed to the latent states, and the inference on the state profiles will become confounded by the extraneous variance.

While the MI provides an important ground for modeling longitudinal variance, the assumption becomes less tenable when it comes to assessment data. In educational and psychological assessments, items are purposefully designed to exhibit distinct psychometric properties (e.g., easy vs. difficulty items, facile vs. laborious tasks) so they can measure various levels of latent traits. The distinct measurement properties of items, if not properly addressed, can bring about systematic variance to outcome data and interfere with the inference on the model parameters and state profiles.

The perceived importance of MI in LMM led to a number of studies exploring ways to test measurement (non)invariance (Di Mari et al., 2022; Kim et al., 2023; Nagelkerke et al., 2016). These studies, however, suggested heuristic approaches comparing model fit or approximating MNI through random effects. Given that assessment data are expected to inherently exhibit MNI, a formal model that explicitly takes into account measurement effects will lend greater utility.

The purpose of this study is to present a refined LMM framework that explicitly models items’ measurement effects and performs inference under the apparent violation of MI. We formulate measurement models of LMM allowing event-specific measurement effects and present an inferential scheme that affords inference on the measurement parameters. The measurement models are formulated for computer-interactive assessments that yield structured log data where items serve as measurement stimuli (e.g., item performance scores, item interaction times, and item action counts). The new inferential scheme is derived from the established analytic solutions (e.g., Baum et al., 1970; Dempster et al., 1977; Rabiner, 1989) while allowing for item-level measurement effects and accommodating different indicator variables (e.g., nominal, ordinal, continuous, count).

The proposed refinement can enhance the functionality of LMM in addressing the MNI and can be applied to any indicator data that exhibit distinct measurement properties (e.g., different indicator categories, distributional characteristics, psychometric qualities). With the inference routines directly derived from the established solutions (e.g., Baum–Welch and Viterbi algorithms), the new framework will demonstrate high implementational efficiency without requiring exploratory model comparison or variance approximation. In this study, we especially consider a computer-interactive assessment for an example application and show that the suggested framework can entertain large-scale multimodal cross-sectional time-series data of many measurement events. The framework is applied to temporal observations from behavioral and cognitive indicators that treat items as measurement events, and it is found that the framework performs reliably well in accounting for items’ measurement effects and decoding underlying latent state profiles.

We emphasize that the focus of the present study is on the methodological refinement of LMM and the demonstration of its empirical performance in noninvariant measurements. While studies exist that modeled measurement effects within a mixture of latent-trait and latent-state models (e.g., Molenaar et al., 2016, 2019; Vermunt et al., 2008), no concrete methodological scheme has been yet established for conducting regular transition analysis in the measurement noninvariant data. The current study presents model formulations and inferential algorithms for implementing LMM in the measurement noninvariant data and demonstrates their experiential performance and relevance to educational assessment data.

In what follows, we present the new LMM framework that relaxes the MI constraint and the corresponding inferential methods that accommodate differential measurement effects. We then report simulation studies that evidence the reliability of the inferential scheme and document the functioning of the modeling framework from numerical experiments. In evaluating the empirical performance, we especially focus on the performance in describing the measurement-variant and invariant data to gauge the validity of the framework, and examine probable consequences of model misuse. The ensuing Section 5 presents an example application of the framework to a real assessment and discusses practical relevance. The article concludes in Section 6 with a summary of findings and directions for future research.

## 2 Modeling framework

### 2.1 Latent Markov model

To establish an LMM framework that accommodates MNI, we consider an assessment setting in which a fixed set of items 
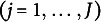
 is administered to a sample of subjects 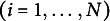 in same sequence. We assume that, at each measurement point *j* (i.e., item assignment), individual’s interaction with the assessment is logged as cross-sectional data that consist of *K* indicators (e.g., response scores, interaction time, action frequency).^1^ Let 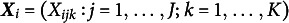 denote a sequence of interaction data observed from a subject *i*. The goal of LMM is to elicit a sequence of latent states, 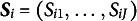 , that explains the emission probabilities of the manifest outcomes. The state variable at each measurement point takes a discrete value from a finite set, 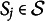 , and is assumed to follow a first-order Markov process, (1)

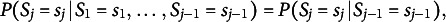

with a realized value, 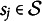 
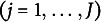
 .^2^ The equality (1) implies that a latent state at any measurement point is determined exclusively by the immediately preceding state and is conditionally independent of the past states.

#### 2.1.1 Structural model

Assuming a latent Markov chain for manifest data necessitates a structural model that describes the behavior of latent states. In LMM, the structural model is formulated by two constituting models: (i) the initial state model that describes the probabilities of initial latent states and (ii) the transition model that describes the transition probabilities of latent states at adjacent event times. Both models can be formulated according to the needs of data. In this study, we apply ordinary multinomial logistic regression and freely estimate state probabilities without particular structure (i.e., stationary state transition, within-state homogeneity).

The initial state model is formulated as (2)

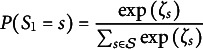

for each 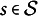 with the state-specific multinomial intercept, 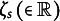 . The state transition model is similarly formulated as (3)

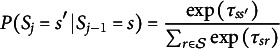

for each 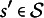 , where 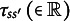 models the logit change from state *s* to 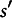 . Models (2) and (3) provide a basic form of the structural model and will be used as a baseline throughout the study. Although not pursued in this study, the models can be extended to integrate covariates (Bartolucci et al., 2014, 2015; Vermunt et al., 1999) or random intercepts (Altman, 2007; Kang et al., 2024; Tang, 2024) or to allow time-variant transitions (Farcomeni, 2015).

#### 2.1.2 Measurement model

Along with the structural model, LMM employs a measurement model to link the observable indicators with the state variables. The model describes emission probabilities of indicator outcomes for each latent state. In this study, measurement models are formulated for the variables that are commonly observed from computer-logged interaction data (e.g., response scores, interaction times, and behavioral counts). The models can accommodate variables in canonical forms. For the variables that exhibit unique distributional characteristics (e.g., skewness, inflated zero counts, and covariate effects), we leave the modification to future work.

For modeling nominal indicators, the study applies multinomial logistic regression (McCullagh & Nelder, 2018). Let 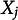 denote a nominal indicator that takes 
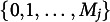
 values. The probability of observing each score value is evaluated as (4)

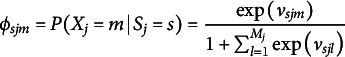

for 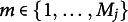 , and 

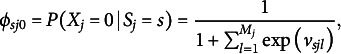

where 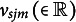 denotes the log odds of a score *m* over a zero score, i.e., 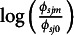 . Observe that the response probabilities are defined for each item (i.e., measurement event) and allow for within-state MNI.

The measurement model for ordinal categorical variables is constructed by implying stochastic ordering between the categories. In this study, we apply an adjacent-categories logit model that connects to the multinomial model (Agresti, 2014, chapter 8; Tutz, 2022). Continuing with the established notation, let 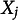 denote an ordinal categorical variable that takes 
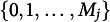
 values. The probability of observing each score value is evaluated as (5)

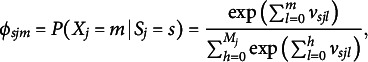

where 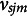 denotes the logit of a response probability between adjacent categories, 
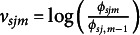
 .

The continuous and count indicator variables are similarly modeled by the canonical models, each with the Gaussian distribution and Poisson regression: (6)

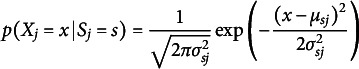

and (7)

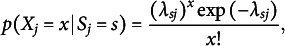

where 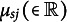 , 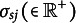 , and 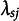 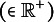 each denote the state-specific location, scale, and rate parameters for item *j*.

#### 2.1.3 LMM

Integrating the constituting models, LMM is defined by the joint probability distribution of 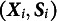 : (8)

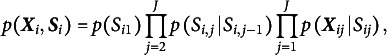

where 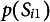 denotes the probability of an initial latent state, 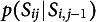 denotes the probability of transitioning latent states from time 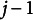 to *j*, and 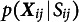 denotes the probability of emitting measurement outcomes, 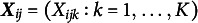 , at state 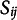 . For notational convenience, we let 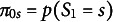 for each 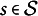 , and 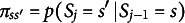 for any 
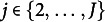
 and 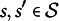 . We again note that both the measurement and structural models can be extended to include covariates as appropriate. This direction is not pursued in this study as our primary interest is in the extension of the LMM framework that accommodates measurement effects.

### 2.2 Inference

Continuing with the assessment setting in which a fixed set of items is administered to a sample of subjects, the parameters of LMM are estimated from multiple chains of multimodal time-series data. Let 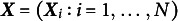 denote a collection of indicator data observed from a calibration sample and 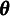 contain the parameters of LMM. The parameter set, 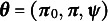 , includes the vector of initial state probabilities, 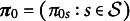 , the state transition probability matrix, 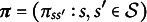 , and the emission parameters of the measurement model, 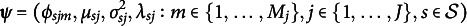 .

Given a set of latent state sequences, 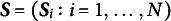 , the likelihood of 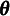 is evaluated by the joint probability distribution of 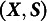 : 

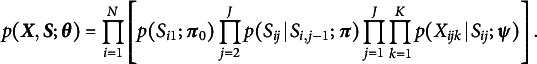

The parameters of LMM are then estimated as the mode of the joint likelihood: 

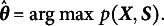



In real settings, the state sequence variable cannot be directly observed and remains latent. To deal with the missing 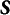 , the expectation–maximization algorithm (Dempster et al., 1977) is employed that iteratively maximizes the conditional expectation of 
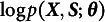
 given the posterior distribution of 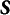 . The algorithm alternates between the expectation and maximization steps to iteratively update the parameters of the model. At the expectation step, the algorithm evaluates conditional expectation of the complete-data log-likelihood based on the provisional estimate, 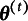 :(9)

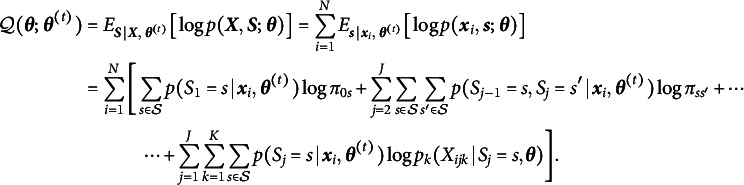



The equation (9) requires computation of posterior probabilities of the unobservable variables, 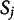 and 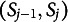 . A practical approach to dealing with the missing state variables is to apply the Baum–Welch (BW) algorithm (Baum et al., 1970). The algorithm draws possible trellis of state paths and applies dynamic recursion programming to evaluate the probabilities that lead up to each state sequence scenario. The original algorithm is designed for measurement-invariant data. In this study, we refine the BW algorithm to accommodate differential measurement effects. We note that the refined estimation bears a resemblance to the process applied in Vermunt et al. (2008). The existing work is aimed for mixture LMMs on categorical outcomes while the present estimation is aimed for standard transition analysis on multimodal indicator data.

#### 2.2.1 Baum–Welch algorithm

Designed for LMM, the BW algorithm applies dynamic recursion programming to compute cardinal probabilities that can estimate 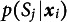 and 
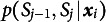
 . Let 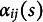 and 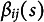 each denote the joint probability of 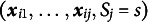 and the conditional probability of 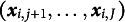 given 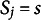 . Each probability measure is evaluated by a recursion algorithm based on the latest parameter values, 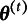 . The joint probability measure, 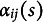 , is obtained via forward recursion as

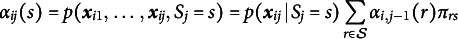

for each item *j* 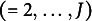 with 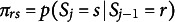 , and 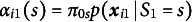 . The conditional probability measure, 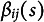 , is obtained via backward recursion as

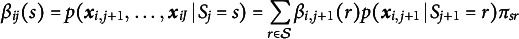

for each *j* 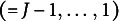 with 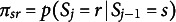 and 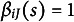 .

The posterior probability of 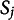 given 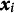 is then evaluated as

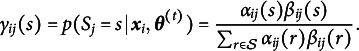

The joint posterior probability of 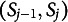 given 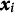 is evaluated as

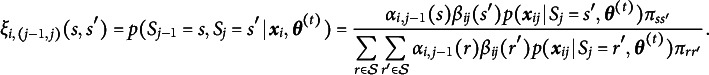

Plugging the posterior probabilities into (9), the 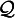 -function becomes 

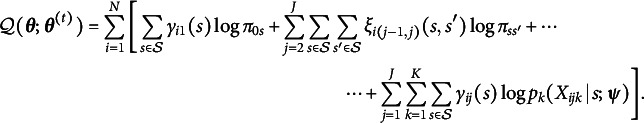



#### 2.2.2 Parameter update

Once the 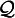 -function is evaluated based on the expected posterior probabilities, the model parameters can be updated via a standard Newton’s method. At the maximization step, the following objective function is maximized to obtain the new parameter iterate: (10)

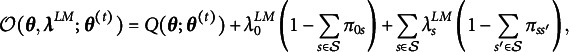

where 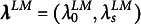 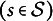 contains Lagrange multipliers that constrain the probability measures.^3^ The 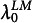 places an equality constraint on the initial state probabilities, 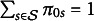 ; each 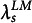 places an equality constraint on the transition probabilities, 
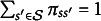
 . The model parameters are then updated as a set of values that maximize the 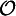 -function: 

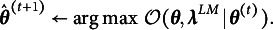



#### 2.2.3 Computation

Equating the score function of (10) at zero yields closed-form equations for some model parameters, allowing analytic computation. For example, the initial state probabilities and state transition probabilities can be updated as 

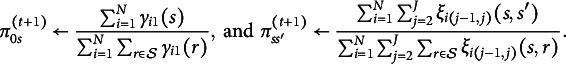

The measurement parameters for the continuous and count outcomes can be updated as 

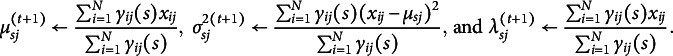

The measurement parameters of the discrete outcomes require numeric iteration and can be updated as the root of the score function: 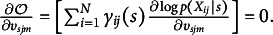


#### 2.2.4 Standard error

As the model parameter estimates are obtained from the above estimation, standard errors can be evaluated based on the Hessian matrix. The standard error of each parameter estimate is obtained as the square root of the diagonal entry of the inverse of the negative Hessian matrix. Supplementary Section A provides the estimating equations for evaluating standard errors of the model parameter estimates.

### 2.3 State estimation

Once the model parameters are estimated with adequate precision, latent states underlying the indicator sequence can be decoded based on the estimated model parameter values. In this study, we obtain state estimates as the most probable state sequence from the posterior probability distribution (i.e., maximum a posteriori). For a subject with the observed data, 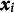 , the state sequence is estimated as (11)

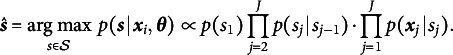

Equation (11) can be solved by the Viterbi algorithm (Rabiner, 1989; Viterbi, 1967) that recursively finds the most probable sequence of latent states. The algorithm evaluates probabilities of possible state sequences that could have generated the indicator sequence and retrospectively determines the most likely sequence of latent states leading to the final state. For implementational details, we refer to Jurafsky and Martin (2019, pp. 152–154, 555–557).

## 3 Simulation study I: Validation

We illustrate the performance of the proposed methods through a series of Monte Carlo simulation studies. In Study I, we verify the accuracy and reliability of the inference scheme and examine the performance of the new LMM framework in the measurement-noninvariant data. In Study II, the new modeling framework is compared with the existing framework in describing the measurement-invariant and noninvariant data.

### 3.1 Design

The performance of the new LMM framework was validated under five-factorial experimental design. The design factors include: (i) latent dimensionality ( 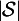 ), (ii) the shape of the initial state probability distribution ( 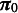 ), (iii) stability of state transition ( 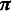 ), (iv) between-state distinction in the emission parameters ( 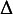 ), and (v) the sample size (*N*). The latent state dimensionality determines the complexity of data and can influence the estimation precision. In this study, the state dimension was varied at 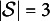 and 5 to simulate moderately and fairly complex latent structures. The state probabilities determine the sample characteristics and can likewise influence the inference outcomes. The present study considered different scenarios for simulating the state probabilities. For the initial state probability distribution, we considered two scenarios—when the distribution is (i) balanced ( 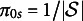 for each 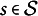 ) and skewed ( 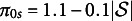 for the first state and 0.1 otherwise; i.e., 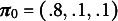 when 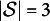 ; (.6, .1, .1, .1, .1) when 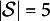 ). The state transition probabilities were similarly simulated for two scenarios: (i) when the states remain stable over time ( 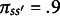 when 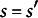 and 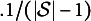 otherwise) and (ii) when the states make moderate transitions ( 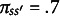 for 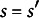 and 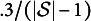 otherwise) (Bacci et al., 2014; Baldwin, 2015).

Along with the structural factors, two additional factors were considered for the measurement model—the difference in the emission parameters between states and the sample size. The between-state difference in the emission parameters dictates distinguishability of the underlying latent states and can influence the overall parameter recovery. In this study, emission parameters were simulated for two scenarios—when the parameters show moderate and large differences (see below for detailed values). The size of calibration data can similarly impact the inference precision and was varied at three levels to create small, moderate, and large sample size conditions— 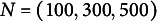 when 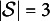 and 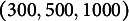 when 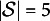 .

The other factors were fixed at constant values. The study assumed a medium-length assessment with 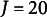 measurement events and collected three indicators at each measurement point ( 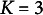 ). The indicator set consisted of ordinal scores (e.g., response scores), continuous outcomes (e.g., reaction times), and count records (e.g., the numbers of erroneous attempts and hints requested). The number of response categories for ordinal outcomes was fixed at four ( 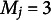 ).

#### 3.1.1 Data generation

Simulating data for LMM yields two data sets: (i) *N*-by-*J* state sequence data and (ii) *N*-by-*J*-by-*K* measurement outcome data. For creating state sequence data, we first obtained initial state variables from the multinomial distribution with 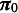 probabilities and generated subsequent state variables from the Markov chains with 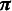 transition probabilities. The measurement outcome data for each latent state were then generated as follows. Among the multiple latent states, we assumed that one state represents a normal test-taking mode and the emission parameters of this state follows a constant set of hyperparameters, 
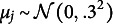
 , 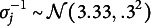 , and 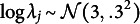 . The emission parameters of other states were generated by shifting the hyperparameters of the normal state as 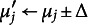 and 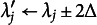 with 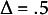 and 1.0 to simulate moderate and large shifts. The parameters of the ordinal outcomes were generated from the uniform distribution, 
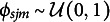
 
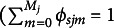
 for each *s*), and reordered according to the hypotheses on the latent states. Table 1 details the scenarios hypothesized for the state labels. When 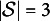 , we assumed that the first state represents the normal test-taking mode, and the other states represent noneffortful and plodding states. When 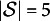 , we assumed that each latent state represents the normal, noneffortful, struggling, efficient, and plodding states.

**Table 1:** Characterization of latent states

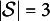	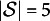
State 1: Normal test-taking mode	State 1: Normal test-taking mode
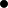 High probabilities of attaining middle accuracy scores: 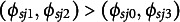	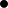 High probabilities of attaining middle accuracy scores: 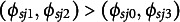
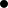 Modest response times: 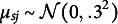 , 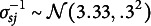	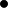 Modest response times: 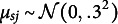 , 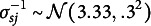
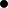 Moderate interactions: 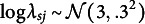	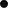 Moderate interactions: 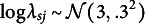
State 2: Noneffortful responding	State 2: Noneffortful responding
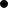 High probabilities of getting low accuracy scores: 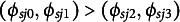	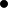 High probabilities of getting low accuracy scores: 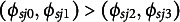
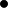 Short response times: 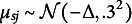 , 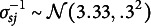	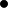 Short response times: 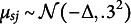 , 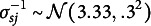
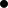 Few interactions: 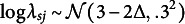	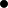 Few interactions: 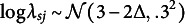
State 3: Plodding	State 3: Struggling
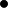 High probabilities of attaining high accuracy scores: 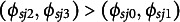	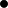 High probabilities of getting low accuracy categories: 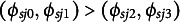
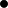 Long response times: 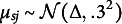 , 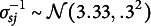	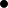 Long response times: 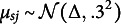 , 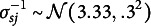
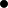 Many interactions: 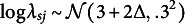	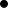 Many interactions: 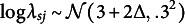
	State 4: Efficient responding
	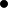 High probabilities of attaining high accuracy scores: 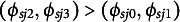
	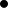 Short response times: 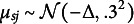 , 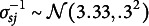
	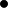 Few interactions: 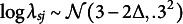
	State 5: Plodding
	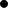 High probabilities of attaining high accuracy scores: 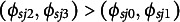
	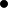 Long response times: 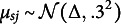 , 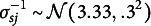
	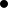 Many interactions: 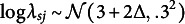

*Note*: 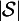 : Number of latent states at each measurement point. 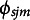 : Probability of responding to category *m* on item *j* at state *s*. 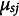 : Mean of log response times on item *j* at state *s*. 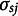 : Standard deviation of log response times in item *j* at state *s*. 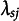 : Mean of action frequencies of state *s* on item *j*. 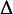 : Degree of shift in the emission parameters; Set at .5 (Moderate) and 1.0 (Large).

As we determine the hyperparameters for each state and obtain emission parameters for each item and state, we generated indicator data following the measurement model formulations. The response score data were generated following (5) on the scale of (0, 1, 2, 3), the response time data following (6) on the log metric, and action count data following (7). All simulation conditions were repeated 100 times each with a unique set of model parameters and calibration data.

#### 3.1.2 Evaluation

While the data simulation yields state sequence data, the state variables cannot be observed in real settings. The inference algorithms for LMM will attempt to estimate the parameters of the model and retrieve the underlying state values. To evaluate the inferential performance of the proposed methods, we estimated the model parameters applying the measurement outcome data and examined the estimation results.

The model estimation was performed applying standard convergence criteria (e.g., log-likelihood tolerance of .001, difference in the iterates less than .005), and the estimation outcomes were evaluated by three measures: (i) bias, (ii) root mean squared error (RMSE), and (iii) standard error. For each model parameter, the bias and RMSE were calculated as 

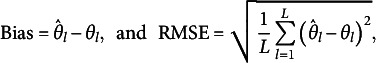

where 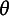 denotes a generating parameter value, 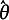 denotes the corresponding estimate, and *l* 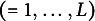 indexes the congeneric parameter (e.g., 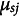 for all 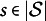 and 
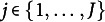
 ).

Since the model parameters were estimated without the knowledge about latent states, a label-switching problem arises when comparing the model parameters. In this study, we determined the order of states based on the proximity of the estimates to the generating parameter values. Among all possible state permutations (e.g., (1, 2, 3), (1, 3, 2), 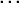 , (3, 2, 1) when 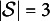 ), the final state set was determined as the one that yielded minimum distance from the generating values (i.e., the most likely state set). In real settings, the label switching is generally not of concern as the true underlying states are not known and the states can be labeled based on the observations from the model parameters.

As we confirm that the model parameters are estimated adequately, we performed state decoding, estimating state sequences underlying the indicator data. The accuracy of the state estimates was evaluated based on the match rate between the estimated and true values: 

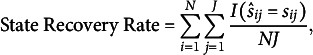

where 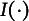 denotes the indicator function, 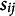 denotes the state value of subject *i* at time *j*, and 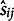 gives the corresponding estimate.

Below we present results of the simulation experiments. The outcomes from the multiple replications were summarized by averaging the evaluation statistics over the repetitions. Where appropriate, partial effect-size measure, 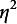 , is reported to inform the significance of the design variables. The value may be interpreted following the convention (Cohen, 1988)— 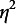 smaller than .01 as a small effect, between .06 and .14 as a medium effect, and larger than .14 as a large effect.

### 3.2 Results

#### 3.2.1 Bias

In Table 2, we report average biases of the parameter estimates from the 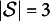 condition. The results for the probability-based parameters were obtained by treating the first state and score levels as a reference category and averaging over the 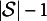 state levels (for 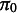 and 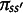 ) and 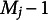 score categories (for 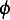 ) to avoid the cancellation due to the sum-to-one constraints (i.e., 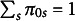 , 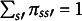 , 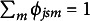 ). The bias results under other reference categories can be found from Supplementary Table B1. The reported values indicate that the estimation overall entailed minimal bias. All parameter domains showed close-to-zero biases with no particular directions. The observed values were constantly small in all evaluation scenarios. All design factors were found to have insignificant impact on the bias statistics, suggesting that the estimation performed stably well against the variation of the design factors.

**Table 2:** Average bias of the model parameter estimates 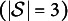

			Balanced initial state distribution						Skewed initial state distribution					
Tr	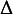	*N*	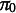	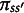	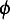	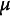	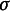	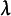	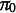	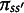	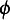	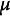	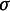	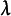
St	Mod	100	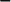 .004	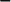 .001	.000	.000	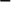 .011	.001	.052	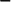 .006	.001	.001	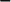 .010	.006
		300	.002	.000	.000	.001	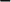 .004	.004	.025	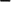 .002	.000	.000	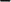 .002	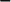 .001
		500	.002	.000	.000	.001	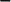 .003	.002	.020	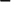 .002	.000	.000	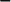 .001	.000
	Lrg	100	.000	.000	.000	.001	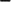 .006	.007	.007	.000	.000	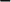 .001	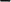 .006	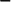 .002
		300	.001	.000	.000	.000	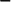 .002	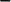 .002	.007	.000	.000	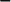 .001	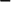 .002	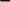 .002
		500	.002	.000	.000	.000	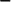 .001	.000	.005	.000	.000	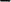 .001	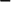 .001	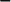 .002
Unst	Mod	100	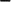 .018	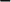 .008	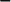 .001	.001	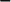 .016	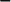 .005	.102	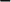 .010	.000	.001	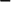 .018	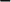 .001
		300	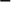 .009	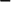 .005	.000	.000	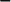 .006	.003	.090	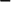 .006	.000	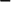 .001	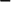 .005	.002
		500	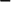 .012	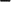 .005	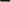 .001	.000	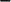 .004	.001	.087	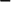 .005	.000	.000	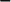 .002	.002
	Lrg	100	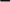 .002	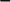 .001	.000	.001	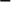 .004	.006	.028	.000	.000	.000	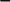 .004	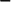 .001
		300	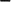 .002	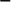 .001	.000	.001	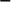 .001	.002	.020	.000	.000	.000	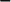 .001	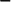 .001
		500	.000	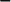 .001	.000	.001	.000	.001	.017	.000	.000	.000	.000	.000

*Note*: Tr: State transition scenarios (St: Stable (stayer probability = .9), Unst: Unstable (.7)). 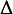 : Difference in the emission parameters (Mod: Moderate (e.g., 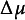 = .5), Lrg: Large (1.0)). *N*: Sample size. 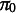 : Initial state probabilities. 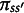 : State transition probabilities. 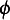 : Response probabilities for ordinal outcomes. 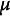 : Location parameter for continuous outcomes. 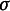 : Scale parameter for continuous outcomes. 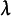 : Rate parameter for count outcomes. The average biases for 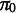 , 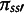 , and 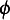 were obtained by treating the first category as a baseline and averaging over the remaining categories.

The results from the 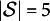 condition showed a similar pattern (see Supplementary Tables B2 and B3). Although the overall magnitude of bias slightly increased due to increased latent complexity, the bias values remained constantly and stably small across the evaluated conditions.

#### 3.2.2 RMSE

In Table 3, we report RMSEs of the parameter estimates for 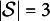 . The reported values again suggest that the estimation performed adequately well in recovering the parameter values. The RMSEs were reasonably small and remained stable across the evaluated conditions. The patterns across the design variables were generally in line with the expectations. An increase in the sample size entailed decrease in the estimation errors and improved parameter recovery ( 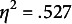 on average). The greater distinction in the emission parameters similarly led to improved recovery ( 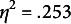 ), reducing RMSEs by .038 on average. The factors related to the state distributions also showed expectable patterns. As the latent states were more evenly distributed and remained stable, the estimation achieved greater accuracy in recovering the model parameters. Between the two evaluated factors, the stability in transition probabilities generally had greater influence on the recovery performance, yielding 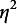 of .087 and an average RMSE difference of .023 between the stable and unstable conditions (vs. 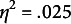 and .009 difference when the initial state probability distribution was varied). All in all, the results from Table 3 evidenced that the estimation performed adequately well and delivered reliable outcomes.

**Table 3:** Root mean squared error of the model parameter estimates 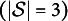

			Balanced initial state distribution						Skewed initial state distribution					
Tr	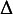	*N*	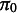	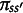	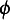	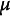	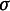	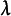	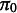	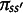	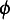	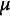	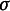	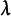
St	Mod	100	.061	.047	.092	.083	.050	.370	.098	.047	.100	.095	.057	.419
		300	.033	.020	.050	.051	.027	.215	.047	.020	.055	.050	.030	.226
		500	.024	.021	.040	.044	.021	.173	.037	.017	.043	.038	.023	.174
	Lrg	100	.043	.010	.077	.055	.040	.306	.032	.010	.084	.066	.049	.348
		300	.024	.006	.044	.032	.023	.174	.022	.006	.048	.037	.027	.196
		500	.021	.005	.034	.024	.018	.135	.017	.005	.037	.028	.021	.150
Unst	Mod	100	.099	.091	.107	.125	.066	.485	.168	.087	.112	.130	.069	.497
		300	.070	.052	.064	.083	.037	.298	.145	.049	.067	.085	.039	.310
		500	.062	.046	.053	.071	.029	.247	.140	.043	.054	.074	.032	.259
	Lrg	100	.049	.017	.079	.062	.047	.331	.057	.017	.081	.072	.053	.345
		300	.026	.010	.045	.036	.026	.189	.037	.010	.046	.041	.030	.195
		500	.022	.008	.034	.028	.021	.146	.030	.008	.036	.032	.024	.151

*Note*: Tr: State transition scenarios (St: Stable (stayer probability = .9), Unst: Unstable (.7)). 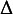 : Difference in the emission parameters (Mod: Moderate (e.g., 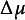 = .5), Lrg: Large (1.0)). *N*: Sample size. 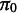 : Initial state probabilities. 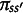 : State transition probabilities. 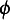 : Response probabilities for ordinal outcomes. 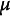 : Location parameter for continuous outcomes. 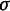 : Scale parameter for continuous outcomes. 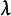 : Rate parameter for count outcomes.

**Table 4:** Latent state recovery rate

	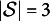					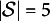				
		Balanced		Skewed			Balanced		Skewed	
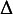	*N*	St	Unst	St	Unst	*N*	St	Unst	St	Unst
.5	100	.888	.752	.882	.749	300	.592	.473	.650	.473
	300	.919	.800	.922	.797	500	.593	.483	.663	.478
	500	.921	.810	.926	.806	1000	.597	.490	.668	.485
1.0	100	.982	.945	.981	.946	300	.641	.605	.702	.623
	300	.984	.951	.983	.953	500	.650	.611	.698	.626
	500	.984	.952	.983	.954	1000	.653	.612	.711	.630

*Note*: 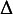 : Difference in the emission parameters (Mod: Moderate (e.g., 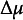 = .5), Lrg: Large (1.0)). *N*: Sample size. Tr: State transition scenarios (St: Stable (stayer probability = .9), Unst: Unstable (.7)).

The RMSE results from the 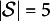 condition showed similar patterns with Table 3 (see Supplementary Table B4). While the estimation errors increased in magnitude as a result of the increase in the free parameters and data complexity, the overall size of errors was acceptably small (.193 on average). The patterns relating to the design variables were also consistent with those in Table 3.

#### 3.2.3 Standard error

In addition to the accuracy criteria, we also evaluated standard errors of the parameter estimates as a measure of estimation precision. Supplementary Table B5 presents detailed results of the evaluated standard errors. As shall be seen from the table, the estimation overall showed a well-regulated behavior in estimating the standard errors. The impact of the design factors was generally negligible ( 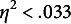 ) except for the sample size ( 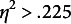 ). As the calibration sample size increased, the estimation consistently yielded smaller standard errors. Besides the sample size, no other design factors showed significant impact, and standard errors remained stable across the evaluated settings.

#### 3.2.4 State recovery

In Table 4, we report match rates between the estimated and true state values. The estimation was performed by the Viterbi algorithm based on the estimated model parameter values. The results suggest that the state estimation overall achieved adequate recovery. When data contained three latent states, the estimation recovered the true states at 90.69% average rate. When there exist five states, the underlying states were recovered at 60.04% rate on average. The estimation performance improved as the calibration data exhibit greater distinction in the emission probabilities ( 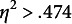 ) and stability in the transition probabilities ( 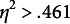 ) (average recovery rate .983 when 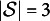 and .676 when 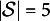 ). The calibration sample size and the shape of the initial state distribution had relatively marginal impact on the estimation of the state profiles ( 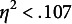 ).

## 4 Simulation study II: Model comparison

As we verify the performance of the new LMM framework, we conducted a second simulation study to evaluate the relative performance of the new framework to the existing model. Applying the measurement-invariant and noninvariant data, we cross-fit the new and existing LMMs and examined the estimation outcomes to gauge the robustness to model misspecification. The outcomes of this experiment can help understand the probable consequences of misusing the models and will illustrate the relative gravity of over- and under-fitting the LMMs.

### 4.1 Design

The simulation settings generally remained analogous to study I except for the data-generating model and latent dimensionality. As the focus of the study shifted to cross-fit performance, we generated data from the two distinct models—the model that assumes MI and the model that allows MNI. The data generated from each model were then cross-fit by the competing model—the MNI model being fit to the measurement invariant data (i.e., overfit) and the MI model being fit to the measurement noninvariant data (i.e., underfit).^4^ Along with the data-generating model, we also adjusted the latent dimensionality of data at 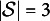 . Simulation study I verified that the estimation routine performs stably across the different latent dimensions. Given this finding, we fixed the latent dimensionality at a constant value and assumed that findings of the present experiment would have similar implications for other dimensions.

**Table 5:** Average absolute bias and state recovery rate of the MNI model fit to the MI data

			Model parameter recovery												State	
			Balanced initial state distribution						Skewed initial state distribution						recovery	
Tr	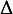	*N*	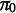	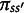	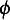	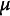	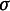	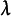	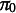	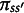	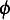	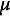	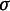	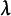	BL	SK
St	Mod	100	.060	.083	.081	.085	.044	.373	.074	.084	.086	.109	.046	.382	.776	.730
		300	.032	.062	.054	.063	.026	.254	.054	.062	.060	.089	.028	.269	.797	.750
		500	.029	.059	.046	.058	.021	.219	.052	.058	.052	.084	.023	.235	.800	.753
	Lrg	100	.043	.013	.059	.044	.033	.242	.066	.012	.064	.079	.035	.259	.972	.912
		300	.023	.007	.034	.025	.019	.140	.049	.006	.040	.064	.021	.156	.977	.915
		500	.017	.006	.026	.020	.015	.108	.046	.005	.033	.058	.017	.127	.978	.915
Unst	Mod	100	.053	.090	.084	.105	.054	.391	.066	.092	.087	.124	.055	.405	.713	.672
		300	.033	.082	.055	.087	.035	.271	.047	.082	.058	.107	.036	.291	.727	.684
		500	.028	.082	.047	.083	.031	.241	.040	.082	.051	.103	.032	.263	.729	.686
	Lrg	100	.041	.018	.062	.049	.037	.250	.064	.017	.067	.084	.039	.277	.942	.882
		300	.023	.011	.035	.028	.021	.148	.046	.011	.041	.065	.023	.178	.950	.887
		500	.018	.009	.027	.022	.016	.113	.043	.009	.034	.060	.019	.146	.951	.888

*Note*: Tr: State transition scenarios (St: Stable (stayer probability = .9), Unst: Unstable (.7)). 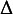 : Difference in the emission parameters (Mod: Moderate (e.g., 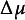 = .5), Lrg: Large (1.0)). *N*: Sample size. 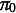 : Initial state probabilities. 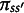 : State transition probabilities. 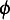 : Response probabilities for ordinal outcomes. 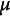 : Location parameter for continuous outcomes. 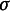 : Scale parameter for continuous outcomes. 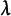 : Rate parameter for count outcomes. BL: Balanced initial state distribution. SK: Skewed initial state distribution. The number of latent states was fixed at 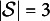 .

**Table 6:** Average absolute bias and state recovery rate of the MI model fit to the MNI data

			Model parameter recovery												State	
			Balanced initial state distribution						Skewed initial state distribution						recovery	
Tr	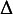	*N*	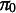	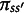	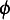	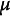	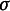	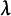	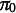	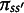	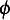	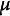	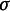	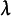	BL	SK
St	Mod	100	.131	.062	.067	.236	.106	.255	.156	.091	.068	.237	.098	.263	.836	.812
		300	.116	.055	.065	.234	.109	.248	.147	.074	.066	.236	.102	.253	.843	.827
		500	.113	.055	.065	.234	.109	.245	.151	.076	.065	.236	.101	.251	.846	.827
	Lrg	100	.049	.011	.065	.233	.115	.236	.040	.011	.065	.230	.112	.239	.970	.970
		300	.036	.007	.064	.232	.116	.231	.031	.007	.064	.230	.113	.235	.971	.970
		500	.035	.006	.064	.232	.116	.230	.028	.006	.064	.230	.114	.234	.971	.970
Unst	Mod	100	.161	.144	.068	.243	.066	.252	.172	.140	.068	.242	.065	.261	.706	.702
		300	.151	.150	.066	.243	.064	.245	.170	.145	.067	.242	.064	.254	.707	.702
		500	.149	.150	.066	.242	.064	.242	.166	.145	.066	.241	.063	.252	.707	.703
	Lrg	100	.073	.018	.064	.236	.121	.240	.046	.017	.065	.230	.111	.241	.919	.924
		300	.062	.014	.063	.236	.120	.236	.041	.012	.064	.229	.112	.236	.919	.924
		500	.061	.012	.063	.236	.121	.235	.040	.011	.064	.229	.112	.235	.920	.924

*Note*: Tr: State transition scenarios (St: Stable (stayer probability = .9), Unst: Unstable (.7)). 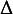 : Difference in the emission parameters (Mod: Moderate (e.g., 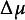 = .5), Lrg: Large (1.0)). *N*: Sample size. 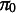 : Initial state probabilities. 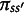 : State transition probabilities. 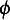 : Response probabilities for ordinal outcomes. 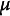 : Location parameter for continuous outcomes. 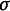 : Scale parameter for continuous outcomes. 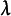 : Rate parameter for count outcomes. BL: Balanced initial state distribution. SK: Skewed initial state distribution. The number of latent states was fixed at 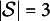 .

#### 4.1.1 Evaluation

The performance of the models was evaluated based on the similar criterion measures with study I while additionally considering absolute bias as a summary measure of biasedness and accuracy. For ease of discussion, we focus on the absolute bias in this article and present other results in Supplementary Material (see Supplementary Tables B8–B10). As with study I, all simulation conditions were repeated 100 times, each with a unique set of parameters and data, and outcomes were summarized by averaging over the replications.

### 4.2 Results

In Tables 5 and 6, we report average absolute biases observed from the two modeling scenarios. The results are presented for the ill-fitted models to compare with the outcomes from the normal fitting (see Supplementary Tables B6 and B7). Comparison of Tables 5 and B6 suggests that assuming heterogeneous measurement effects in the invariant data can lead to increased estimation error. As the MNI model was fit to the invariant data, the model parameter estimation yielded .034 larger absolute bias and .027 lower state recovery rate compared to the normal fitting. The increase in the estimation errors was especially pronounced when data contained few observations (.053 increase in absolute bias on average) and displayed greater distinction in the emission parameters (.042 increase in absolute bias and .035 decrease in state recovery rate). The increased disparity in these settings, however, appeared to be due to the relatively stable performance of the MI model rather than of the poor performance of the MNI model. The MI model showed comparatively strong performance in the above-described settings, making the contrast with the MNI model more distinct. When evaluated the absolute performance of the MNI model under the same settings, estimation errors were reasonably small, showing .106 average absolute bias when 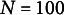 and .058 average absolute bias and .931 average state recovery rate when the emission parameters were of large distinction.

Comparing Table 6 and Supplementary Table B7 similarly illuminates consequences of underfitting. When the MI model was fit to the measurement-noninvariant data, the model parameter estimates came to contain larger errors (.069 larger absolute bias on average) and the state profiles were less likely to match the true values (.050 lower recovery rate on average). The impact of underfitting was especially dire in large calibration data. In normal fitting, the increase in the sample size resulted in smaller estimation errors ( 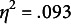 on average). When the model was underfit, however, increasing the sample size had a marginal influence ( 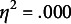 ) and the estimation errors remained constantly large across the different sample conditions.

Compared with the findings from Table 5, the trends in Table 6 seemed to imply that constraining measurement parameters in the measurement-noninvariant data can lead to larger estimation errors than overfitting and that the increased errors are harder to rectify. For example, if the MNI model is mistakenly fit to the measurement-invariant data, increasing the sample size ( 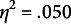 ) can help remedy the increased errors and attain outcomes comparable to the MI model (e.g., .021 absolute bias difference and .027 state recovery difference when 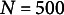 ). If the MI model is fit to the measurement-noninvariant data, on the other hand, no other adjustments or manipulations can be made to remedy the superfluous errors caused by the misfit.

## 5 Real data application

The performance of the proposed methods was further examined using empirical data from an international educational assessment, the Program for International Student Assessment (PISA). PISA measures 15-year-old pupils’ scholastic ability in reading, mathematics, and science and is known to exhibit large variation in students’ test-taking behaviors due to its low-stakes consequences. In this study, we performed transition analysis on example assessment data and examined students’ latent mental processes during the assessment.

### 5.1 Analysis setting

#### 5.1.1 Data

The example data were obtained from the 2015 Science assessment, the last assessment administered in linear forms. Science was the main subject area in 2015 and was chosen for its extensive sample data. For example analysis, we drew out S07-12 booklets that were newly released in the administration year and performed transition analysis on the US sample data. The examined data contained 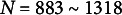 observations after cleaning (students with more than five missing entries were removed).^5^ The number of items (i.e., the number of measurement points) ranged between 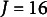 and 18 with a majority of items being scored dichotomously and a few scored polytomously on the scale of (no, partial, full) credits. The items on the booklets varied in presentation forms (e.g., simple multiple choice, complex multiple choice, open responses) and showed distinct patterns in the interaction indicators.

#### 5.1.2 Analysis

The transition analysis was performed based on three interaction indicators: (i) the ordinal response scores, (ii) response times on a log scale, and (iii) the number of total actions on each item. The count outcomes from the examined data showed large variations (e.g., a maximum of 251 actions on a simple multiple-choice item) and were treated as continuous after a log transformation.^6^ For modeling interaction behaviors, we applied the two forms of LMM: (i) the model that assumes MI and (ii) the model that allows MNI. Note that the MI model does not allow variation in the measurement stimuli and cannot be applied to the raw data that differ in the number of response categories. For evaluating relative performance of the models, the data needed to be reshaped so that both the models can be applied. In this study, we created factitious data that dichotomized the raw score data into two categories (0: no/partial, 1: full credits) to compare the performance of the models and additionally applied the original set of data to evaluate the performance of the MNI model in the raw outcomes.

#### 5.1.3 Evaluation

The comparison of the models was performed based on the relative model fit measures, including the Akaike information criterion (AIC; Akaike, 1973), corrected AIC (Burnham & Anderson, 2002; Sugiura, 1978), Bayesian information criterion (BIC; Schwartz, 1978), and adjusted BIC (Sclove, 1987).^7^ The same set of criterion measures was used to determine the number of latent states underlying the data (Bartolucci et al., 2017). Below we present results and findings from the empirical analysis. For ease of discussion, results are presented for two booklets that showed distinct patterns.

### 5.2 Analysis I: Booklet S07

Table 7 reports fit statistics of the models applied to the S07 booklet data ( 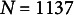 , 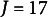 ).^8^ The models were fit to the two data sets: (i) the original set of data that contain ordinal response scores, log interaction times, and log action counts, and (ii) the recoded data that contain dichotomized response scores and process indicators. The fit results of the MI model for the raw data are missing because the model does not allow variation in the item characteristics and could not be applied to the original data that differ in the response categories.

**Table 7:** Relative model fit statistics from the S07 booklet data

Data	Mod	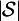	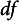	logLike	AIC	CAIC	BIC	ABIC
Recoded	MI	1	5	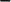 70930.5	141866.1	141901.3	141896.3	141880.4
		2	13	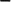 61371.5	122755.9	122847.4	122834.4	122793.1
		3	23	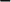 61286.7	122596.4	122758.3	122735.3	122662.2
		4	35	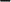 58772.4	117579.9	**117826.1**	**117791.1**	**117680.0**
		5	49	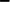 **58747.7**	**117544.4**	117889.2	117840.2	117684.6
	MNI	1	85	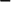 45078.9	90242.8	90840.9	90755.9	90485.9
		2	173	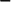 42384.3	84989.6	**86544.6**	**86323.6**	**85621.7**
		3	263	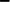 **42076.2**	**84543.5**	87294.6	86903.6	85661.7
		4	355	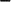 44483.4	89561.8	93748.3	93153.3	91263.4
		5	449	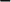 44377.3	89587.6	95448.7	94615.7	91969.9
Raw	MNI	1	88	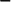 46493.2	93074.5	93693.6	93605.6	93326.1
		2	179	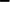 **43345.4**	**86917.8**	**88515.0**	**88288.0**	**87567.0**
		3	272	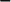 43426.0	87252.0	90066.4	89666.4	88395.9
		4	367	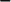 45396.9	91400.8	95671.7	95064.7	93136.7
		5	464	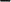 46097.9	93043.7	99010.4	98162.4	95468.9

*Note*: Data: Recoded (Dichotomous response scores, Log interaction times, Log action counts); Raw (Polytomous response scores, Log interaction times, Log action counts). Mod: Model (MI: Measurement invariance, MNI: Measurement noninvariance). 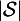 : Number of states. 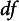 : Degrees of freedom. logLike: log-likelihood. AIC: Akaike information criterion. CAIC: Corrected AIC. BIC: Bayesian information criterion. ABIC: Adjusted BIC. The best outcomes under each condition are boldfaced.

**Table 8:** Average emission parameter values in the S07 booklet data

	Response score			Interaction time		Number of actions	
	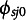	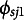	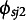	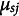	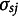	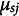	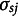
Simple MC							
State 1	.669 (.045)	.331 (.030)	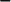	3.288 (.047)	.836 (.033)	1.130 (.026)	.485 (.018)
State 2	.278 (.019)	.722 (.031)	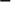	3.781 (.019)	.487 (.013)	1.086 (.016)	.415 (.012)
Complex MC							
State 1	.462 (.038)	.493 (.036)	.317 (.030)	3.541 (.037)	.621 (.026)	1.668 (.016)	.256 (.011)
State 2	.308 (.019)	.655 (.028)	.263 (.018)	4.121 (.017)	.477 (.012)	1.923 (.014)	.391 (.010)
Open Response							
State 1	.889 (.056)	.095 (.016)	.041 (.014)	3.866 (.052)	.859 (.037)	2.736 (.080)	1.313 (.057)
State 2	.465 (.023)	.405 (.021)	.324 (.020)	4.791 (.014)	.423 (.010)	4.577 (.019)	.547 (.013)

*Note*: MC: Multiple choice. 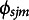 : Probability of scoring *m* on item *j* at state *s*. 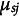 : Mean of the continuous outcome of item *j* at state *s*. 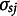 : Standard deviation of the continuous outcome of item *j* at state *s*. Within the parentheses are average of the standard errors of the parameter estimates.

In Table 7, the results from the recoded data suggest that the MNI model overall achieved a better fit than the MI model. While the model increased in the number of free parameters, it constantly yielded smaller criterion statistics when the latent dimension was held constant. The MI model on the other hand showed much lower likelihood and demonstrated constantly subpar fitness compared to the MNI model.

The fit statistics within each model also suggested distinct patterns related to the latent dimensionality. In the MI model, the fit measures achieved the best outcomes when the number of latent states was conditioned at four or five. In the MNI model, the measures showed the best performance as the dimension was set at two or three. When applied to the raw outcome data, the MNI model similarly favored the two-state solution, consistently suggesting fewer latent dimensions than the MI model. Provided that the MI model does not allow variance in the measurements, it seemed that the model tended to ascribe residual variance from the items to the latent factors and came to overpredict the underlying latent dimensionality. All in all, the comparison of the fit statistics in Table 7 suggested that the MNI model better describes the observed data and the variation in the indicator variables can be reasonably summarized by two latent states. In the following discussion, we elaborate findings from the two-state MNI model.

The state probability estimates from the two-state MNI model suggested that a majority of students entered the booklet in State 1 (62.63%) and showed a strong tendency to stay in the same state across the assessment (.700 (State 1), .911 (State 2)). Students in State 1 tended to receive low accuracy response scores (.415 on average (SD = .533)), spend little time on the items (50.154 seconds on average (50.791)), and attempt a few interactions (11.643 actions on average (31.633)). Students in State 1 generally attained higher accuracy scores (.713 (.558)), spent more time on the items (85.068 (62.021)), and exerted more interactions (50.999 (96.804)). Taking the patterns from the indicators together, it could be inferred that State 1 represents a state of less attention and less effort and State 2 of greater attention and more effort.

The emission parameter estimates from the model indicated a consistent finding with the observations from the state estimates and the raw data. In Table 8, we report average measurement parameter values for the different state levels and item types (see Supplementary Table B11 for item-level estimates). The emission parameters from State 1 were consistently associated with the larger probabilities of low response scores, lower time intensities, and fewer action counts. The parameters from State 2 were associated with the larger probabilities of high response scores, longer times, and greater interaction efforts. The parameter values from the different item types were also found consistent with the expectation. The simple multiple-choice (MC) items tended to involve less time and relatively few interactions ( 
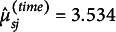
 , 
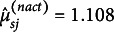
 on average). The complex MC items required greater time efforts and interactions (each with 3.831 and 1.796 on average). The open-response items were associated with the greatest intensities in time and action efforts (4.329 and 3.657 each).

### 5.3 Analysis II: Booklet S09

Among the six booklets examined, four booklets 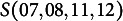 indicated two latent states and the other two 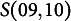 three states. Below we present outcomes from the S09 booklet ( 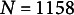 , 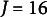 ) that showed the other distinct patterns.

Table 9 reports fit statistics of the models fit to the two sets of trimodal data: (i) the recoded data that contain binary response scores, log interaction times, and log action counts and (ii) the raw data with ordinal scores and the time and count values. The results from the table confirm the consistent findings on the models. The MNI model constantly achieved greater fitness when the calibration data and latent dimensions were held constant. The MI model suggested a greater number of latent states due possibly to no avenue for accounting for varying measurement effects of items.

**Table 9:** Relative model fit statistics from the S09 booklet data

Data	Mod	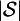	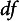	logLike	AIC	CAIC	BIC	ABIC
Recoded	MI	1	5	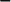 68058.0	136126.0	136126.1	136151.3	136135.4
		2	13	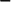 62181.6	124389.2	124389.5	124454.9	124413.6
		3	23	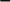 60465.9	120977.7	120978.7	121094.0	121020.9
		4	35	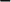 59526.5	119123.1	119125.3	119300.0	119188.8
		5	49	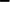 **59438.7**	**118975.4**	**118979.8**	**119223.0**	**119067.4**
	MNI	1	80	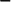 43638.4	87436.8	87448.9	87841.2	87587.1
		2	163	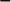 43461.0	87248.1	87301.9	88072.0	87554.2
		3	248	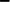 **41305.1**	**83106.1**	**83242.0**	**84359.6**	**83571.9**
		4	335	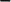 43214.6	87099.2	87373.1	88792.4	87728.4
		5	424	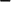 47622.4	96092.8	96584.4	98235.9	96889.1
Raw	MNI	1	81	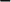 44303.3	88768.6	88781.0	89178.0	88920.7
		2	165	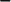 44039.1	88408.1	88463.4	89242.1	88718.0
		3	251	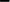 **42207.9**	**84917.9**	**85057.5**	**86186.5**	**85389.3**
		4	339	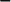 43596.9	87871.9	88153.7	89585.3	88508.5
		5	429	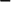 44565.9	89989.8	90496.6	92158.1	90795.5

*Note*: Data: Recoded (Dichotomous response scores, Log interaction times, Log action counts); Raw (Polytomous response scores, Log interaction times, Log action counts). Mod: Model (MI: Measurement invariance, MNI: Measurement noninvariance). 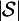 : Number of states. 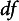 : Degrees of freedom. logLike: log-likelihood. AIC: Akaike information criterion. CAIC: Corrected AIC. BIC: Bayesian information criterion. ABIC: Adjusted BIC. The best outcomes under each condition are boldfaced.

Examining outcomes of the three-state MNI model revealed that students tended to begin the booklet in States 2 (54.48%) and 3 (34.90%) and stay in the same state. The staying and transition probabilities were estimated as 

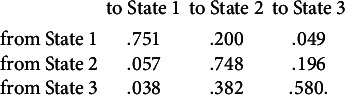

The patterns in the indicator variables suggested that students in State 1 tended to receive low accuracy scores (.117 on average (SD = .321)), devote little time (42.246 seconds on average (50.301)), and show relatively few actions (10.819 actions on average (35.930)). Students in State 2 attained medium scores (.432 (.502)) with time and interaction efforts in middle ranges (64.625 seconds (38.444); 30.255 actions (47.895)). Those in State 3 received relatively high scores (.450 (.544)) and showed distinctly long interaction times (125.089 (78.397)) and many actions (89.992 (148.740)). Taking the indicator patterns collectively, it could be concluded that State 1 represents a less effortful mode, State 2 a conscientious working mode, and State 3 a state of plodding.

The emission parameters presented in Table 10 supported similar conclusions (see Supplementary Table B12 for detailed results). The parameters from State 1 were associated with the larger probabilities of low accuracy scores, and less time and interaction intensities. Those from State 2 were associated with the larger probabilities of high accuracy scores, and moderate intensities of time and interaction efforts. Those from State 3 were associated with the larger probabilities of high accuracy scores and intense interaction efforts. The parameter values for the different types of items showed reckonable trends—the simple MC items entailing less time and interaction intensities ( 
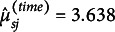
 , 
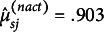
 ), the complex MC items invoking increased interaction efforts (each with 3.840 and 1.852 on average) and the open-response items requiring most intensive problem-solving efforts (4.408 and 3.912 each).

**Table 10:** Average emission parameter values in the S09 booklet data

	Response score			Interaction time		Number of actions	
	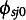	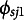	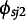	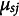	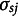	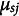	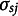
Simple MC							
State 1	.582 (.060)	.418 (.049)	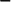	3.206 (.067)	.833 (.047)	.870 (.035)	.447 (.025)
State 2	.456 (.024)	.544 (.028)	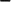	3.667 (.016)	.396 (.011)	.641 (.012)	.309 (.009)
State 3	.469 (.034)	.531 (.038)	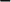	4.040 (.025)	.469 (.018)	1.198 (.029)	.558 (.021)
Complex MC							
State 1	.884 (.068)	.116 (.026)	.000 (.004)	3.338 (.051)	.698 (.036)	1.689 (.029)	.402 (.020)
State 2	.506 (.027)	.486 (.026)	.064 (.010)	3.900 (.013)	.321 (.009)	1.736 (.011)	.276 (.009)
State 3	.548 (.042)	.414 (.037)	.300 (.028)	4.281 (.028)	.469 (.019)	2.132 (.029)	.502 (.020)
Open Response							
State 1	.979 (.074)	.021 (.037)	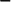	3.666 (.064)	.851 (.046)	2.155 (.087)	1.151 (.062)
State 2	.764 (.035)	.236 (.018)	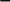	4.458 (.015)	.378 (.011)	4.284 (.022)	.560 (.016)
State 3	.644 (.043)	.356 (.031)	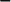	5.099 (.019)	.355 (.014)	5.296 (.025)	.465 (.018)

*Note*: MC: Multiple choice. 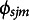 : Probability of scoring *m* on item *j* at state *s*. 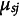 : Mean of the continuous outcome of item *j* at state *s*. 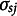 : Standard deviation of the continuous outcome of item *j* at state *s*. Within the parentheses are average of the standard errors of the parameter estimates.

### 5.4 Analysis III: Cluster 20

The calibration results from the booklet data suggested that the MNI model performs reliably well and provides sensible outcomes. In the subsequent analysis, we analyzed cluster-level data to investigate the development of latent processes over a span of time. In PISA, students receive a battery of four booklets—two from the major subject area and two from the secondary subject areas—, and their test-taking behaviors can change across the occasions. In this study, we applied the MNI model to example cluster data to track the evolution of interaction patterns across the two booklets of science assessment.

The example data were obtained from Science Cluster 20 that assigned S07 and S09 as the first two booklets. The data contained 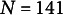 students’ interaction observations on 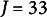 items. As with the above analysis, we used (response scores, interaction times, and action counts) as interaction outcomes and fit the MNI model assuming different latent dimensionalities. The final size of latent dimensions was determined based on the relative model fit measures.

**Figure 1:**
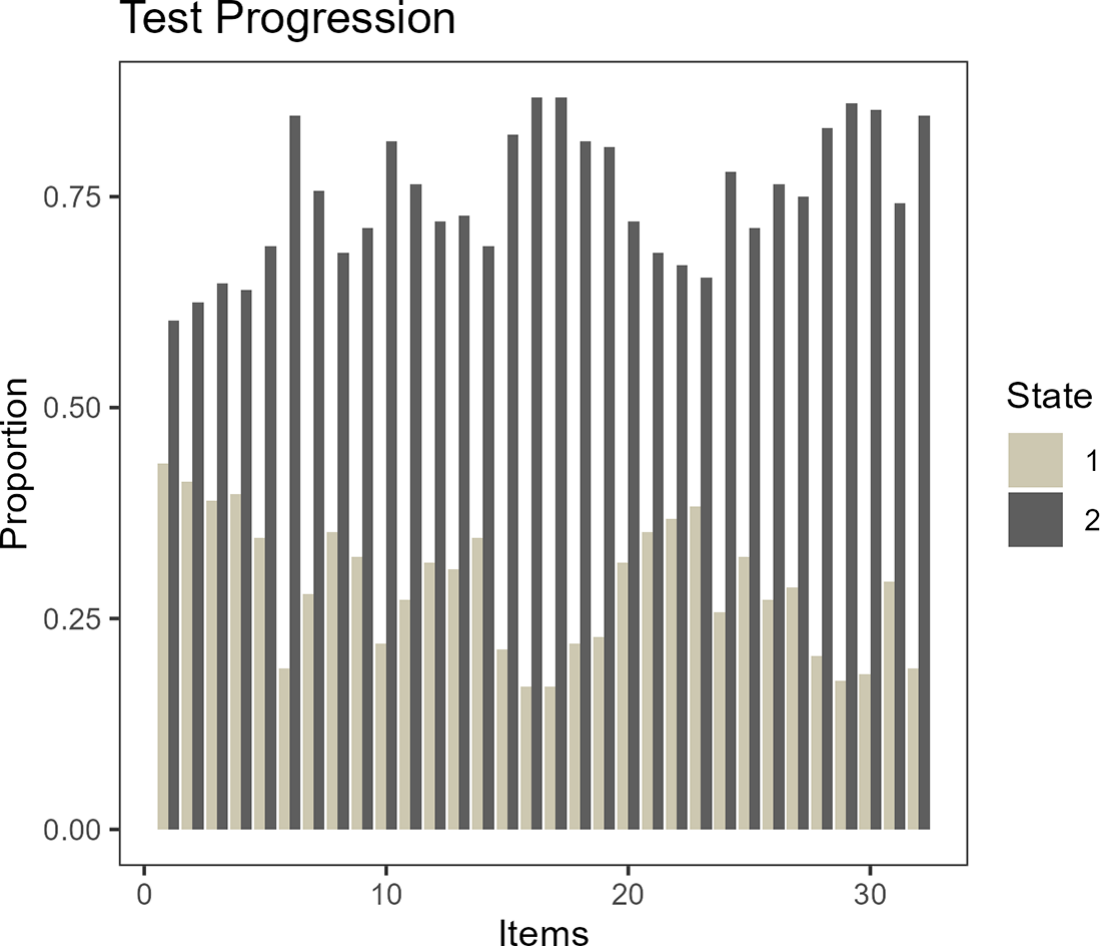
State progression across the assessment. *Note*: State 1 was conceived as a less effortful state and State 2 as a more conscientious state based on the patterns in the indicator variables.

The results from the model suggested a similar pattern to the above analyses. The fit measures consistently endorsed the two-state solution, characterizing State 1 as a less effortful state (i.e., lower accuracy scores, shorter response times, and fewer interactions) and State 2 as a more attentive and conscientiously-working state (i.e., higher accuracy scores, longer interaction times, and many interactions). The state probability estimates from the model suggested that students tended to begin the assessment with approximately equal probabilities of States 1 and 2 (.462 and .538 each) and gradually immerse in State 2 as the assessment progresses ( 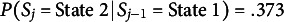 , 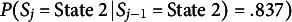 . Figure 1 delineates the prevalence of the states across the assessment stages. As can be seen, a portion of students began the assessment in a less effortful mode and gradually delivered stable performance as the assessment progressed.

**Figure 2:**
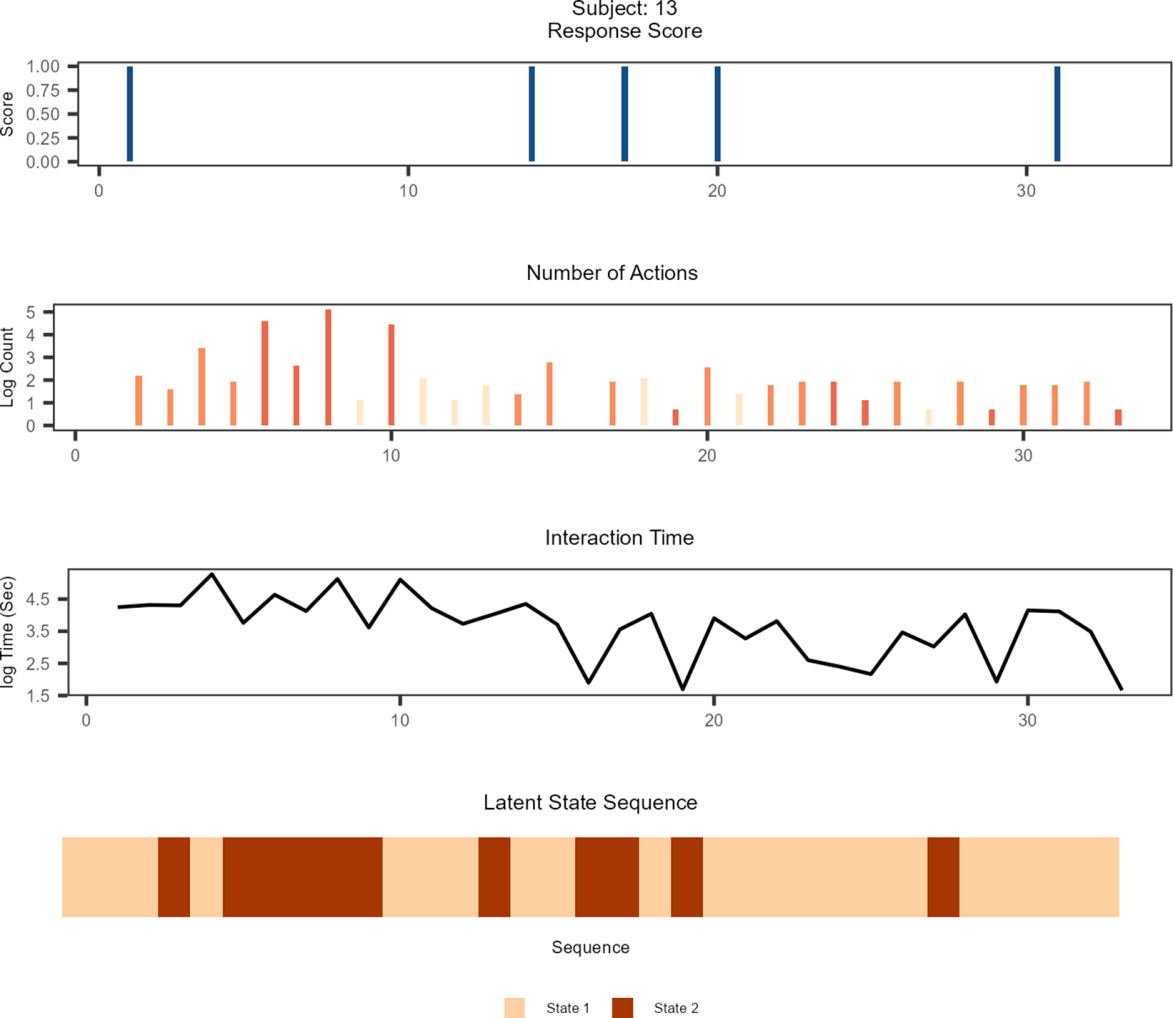
State trajectory of an example student who scored low.

**Figure 3:**
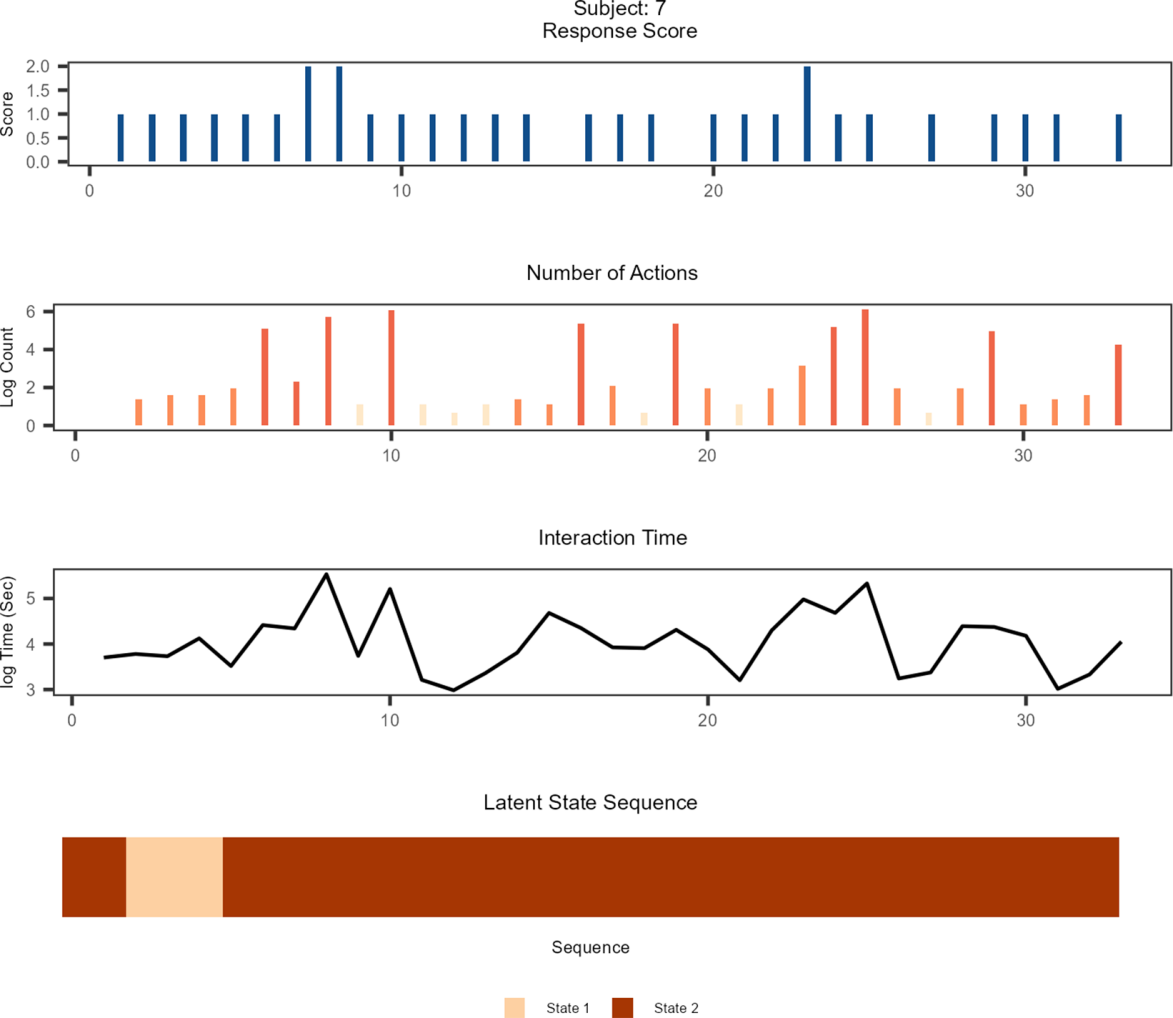
State trajectory of an example student who scored high. *Note*: The brightness of the color in the count outcomes indicates different item types. The brightest color corresponds to simple MC items that involved least interaction; the moderately dark color represents multiple MC items that entailed moderate interactions; and the darkest color represents open-response items that required most intensive interactions.

In Figures 2 and 3, we introduce state trajectories of two example students who attained low ( 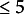 ) and high ( 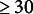 ) total scores. Both figures show that the students displayed moderate variation in their interaction behaviors. As latent states were estimated by the MNI model, distinct patterns were observed regarding the underlying mental process. In Figure 2, the student showed modest activities at the beginning of the assessment and tended to display a retreating behavior as the assessment progressed, resulting in a low total score. The student’s state estimates revealed that the student was indeed in a normal working mode at the beginning but frequently transitioned between the effortful and noneffortful states toward the end of the assessment. The student in Figure 3 similarly showed varying interaction patterns across the assessment, and yet, the patterns closely conformed to the demands of the different item types. In both the figures, the color brightness in the count outcomes indicates different item types—the brightest indicating the simple MC items, the moderately dark color the multiple MC items, and the darkest color representing the open-response items. Figure 3 reveals that whenever the student exerted adequate amounts of efforts that are needed for the items, the student was estimated to be in a normal working mode. On the whole, the students from the low-scoring group showed patterns similar to Figure 2 and those from the high-scoring group similar to Figure 3. Students in the middle-score category tended to show fewer effortful states but more frequent transitions than those in the high-scoring group. All in all, the observations from the state estimates corroborate that the LMM-MNI framework provides sensible state estimates that appropriately take into account the measurement properties of the items.

## 6 Conclusion

The purpose of this study was to present a refined LMM framework that accommodates event-specific measurement effects and performs transition analysis under longitudinal MNI. The traditional LMMs assume that stimuli of measurement products exhibit constant properties and do not contribute to the variance of outcome data. This assumption of MI is not generally tenable in educational and psychological assessments as items exhibit distinct psychometric properties. In this study, we proposed a refinement strategy for LMM that relieves the invariance constraint and accommodates the noninvariant measurements. We formulated the measurement model of LMM, accounting for items’ unique measurement properties, and modified the estimation procedures to enable inference on the measurement parameters. The empirical performance of the new framework was evaluated by numerical experimentation with simulated data and through the application to real assessment data.

The observations from the simulation studies suggest that the new inference procedures perform adequately well in recovering the model parameters and profiling the latent states. The bias and estimation errors were kept small across various validation scenarios. The latent state profiles were accurately identified, showing reasonably high match rates with the true values. The simulation experiments on the whole suggested that the new framework achieves reliable and stable performance while appropriately accounting for variant measurement effects. In addition to the inferential achievement, the performance of the new framework was also evaluated in modeling measurement invariant data. Comparison with the existing model suggested that the new framework carries relatively minor repercussions from overfitting and the inference outcomes remain reliable despite the misspecification. Underfitting the measurement-invariance model to measurement-noninvariant data, on the contrary, led to relatively larger estimation errors and the errors remained persistent across the evaluation scenarios, making it difficult to rectify with the change of environmental factors. Lastly, the empirical analysis of real assessment data suggested that the new framework demonstrates adequate practical relevance and provides credible inference outcomes that align with the observations from the manifest data.

Arguably, the proposed LMM framework achieves greater flexibility in modeling assessment data as it explicitly takes into account the variation in the measurement process and can perform transition analysis in the presence of distinct measurement effects. Refined for interaction log data from computerized assessments, the new framework can accommodate various indicator variables that differ in the measurement level (e.g., nominal, ordinal, continuous, count). The experimental analysis of real assessment data indeed showed that the new framework better addresses the needs of real-life data (e.g., items that differ in the response categories and presentation forms) and demonstrates superior model fit than the traditional measurement-invariance model. The inference procedures that are proposed along with the model were also shown to hold practical value. The numerical results from the simulation studies evidenced that the inference scheme delivers reliable performance even when data contain multiple indicators of many measurement events (i.e., intensive multimodal pooled data). The estimation was achieved with high computational efficiency, affording calibration of large sample data, and extensive replications across multiple validation scenarios.

While the primary focus of this study was on the extension of the measurement model, the LMM framework can be further elevated to enhance the flexibility and applicability. One immediate extension is inclusion of covariates. LMM can accommodate various covariates in different sub-models. For example, subject-level covariates (e.g., demographics) can be included in the structural model to improve the predictability of the state memberships and transitions, or in the measurement model to investigate differential measurement processes across the subgroups. The item- and indicator-specific covariates (e.g., item format, minimum interactions needed) can be similarly added to the measurement model to contemplate relevant research inquiries. The other extension can be made on the transition model. While the present study assumed the conventional first-order time-invariant Markov chain, the transition model can be extended to allow for higher-order Markov processes or time-variant transitions to accommodate the needs of data (e.g., Farcomeni, 2015). Another extension of the LMM framework is an adaptation of the measurement model. As alluded to in Section 2, the measurement models can be adjusted to describe unique distributional characteristics of indicator data (e.g., skewness, zero inflation) or to describe extra variation among subjects (e.g., Altman, 2007; Song et al., 2017). The model inference methods can also be enhanced to tackle missing observations (e.g., Boeschoten et al., 2020; Luo & Du, 2003) or to ensure global optimization (e.g., Do & Artières, 2012). The current refinement of the LMM framework can be easily integrated in the above extensions to further enhance the functionality in serving the needs of real-world applications.

## Supporting information

Kang supplementary material Kang supplementary material

## Footnotes

1 We view interaction outcomes as time series from a sequence of measurement events. For activity stream data that are observed without specific measurement stimuli (e.g., navigation patterns, keystrokes), LMM can be applied after aggregating data (e.g., Maqsood et al., 2022) or after numeric scaling (e.g., Tang et al., 2020, 2021), or at the single-subject level (e.g., Tang, 2024; Xiao et al., 2021).

2 Throughout,  is used to denote the probability of an event and  to denote the probability distribution. The notation,  , will be used to denote the cardinality of the state set (i.e., the number of latent states).

3 The superscript,  , is used to differentiate from the rate parameter,  , of the Poisson distribution.

4 The estimation accuracy of MI was verified prior to the investigation (see Supplementary Table B6).

5 The interaction logs with missing entries were ignored when evaluating the measurement likelihood.

6 The normality of the continuous indicators was evaluated prior to model fitting.

7 The performance of the fit measures was evaluated retrospectively based on the parameter estimates from the real data. See Supplementary Section C for details.

8 The numeric values of the log-likelihood do not monotonically increase along with the increase in the number of states. Due to the challenge in achieving global optima with the expectation-maximization algorithm in LMM, the models were uniformly initialized applying nonparametric clustering (i.e., partitioning around medoids on the Gower distance metric). While the resulting numeric values may not represent global optima of the models, they allow comparison of the models under the controlled setting. See Supplementary Section C for the empirical performance of the log-likelihood and the related information criteria in identifying the number of latent states.

## Supplementary material

The supplementary material for this article can be found at https://doi.org/10.1017/psy.2025.10029.
