# Supplementary material for: A Latent Markov Model for Noninvariant Measurements: An Application to Interaction Log Data From Computer-Interactive Assessments
Source: Psychometrika. 2025 Aug 26;90(4):1481–505. doi: 10.1017/psy.2025.10029 (PMC12660023; doi:10.1017/psy.2025.10029)
Supplement: Kang supplementary material [file S003331232510029Xsup001.pdf]

Appendix A. Standard Error Estimation

The Hessian of each parameter estimate is evaluated as follows. For state probability measures,

$$\frac{\partial^2 \mathcal{O}}{\partial \pi_{0s}^2} = -\frac{1}{\pi_{0s}^2} \sum_{i=1}^{N} \gamma_{i1}(s), \quad \text{and}$$

$$\frac{\partial^2 \mathcal{O}}{\partial \pi_{ss'}^2} = -\frac{1}{\pi_{ss'}^2} \sum_{i=1}^{N} \sum_{j=2}^{J} \xi_{i(j-1,j)}(s,\, s').$$

For nominal outcomes,

$$\frac{\partial^2 \mathcal{O}}{\partial \upsilon_{sjl'} \partial \upsilon_{sjl}} = \begin{cases} -\phi_{sjl}(1-\phi_{sjl}) \sum_{i=1}^{N} \sum_{m=0}^{M_j} u_{ijm} \gamma_{ij}(s) & \text{for } l' = l \\ \phi_{sjl}\phi_{sjl'} \sum_{i=1}^{N} \sum_{m=0}^{M_j} u_{ijm} \gamma_{ij}(s) & \text{for } l' \neq l, \end{cases}$$

where $u_{ijm} = 1$ if the subject i scored m on item j; $u_{ijm} = 0$ otherwise. For ordinal outcomes,

$$\frac{\partial^2 \mathcal{O}}{\partial \nu_{sjl'} \partial \nu_{sjl}} = \begin{cases} -\sum_{i=1}^{N} \gamma_{ij}(s) \left(\sum_{h=0}^{M_j} u_{ijh} \right) \left(\sum_{h=l}^{M_j} \phi_{sjh} \right) \left(1 - \sum_{h=l'}^{M_j} \phi_{sjh} \right) & \text{for } l' \leq l \\ -\sum_{i=1}^{N} \gamma_{ij}(s) \left(\sum_{h=0}^{M_j} u_{ijh} \right) \left(\sum_{h=l'}^{M_j} \phi_{sjh} \right) \left(1 - \sum_{h=l}^{M_j} \phi_{sjh} \right) & \text{for } l' > l. \end{cases}$$

For continuous and count outcomes,

$$\frac{\partial^2 \mathcal{O}}{\partial \mu_{sj}^2} = -\sum_{i=1}^{N} \frac{\gamma_{ij}(s)}{\sigma_{sj}^2},$$

$$\frac{\partial^2 \mathcal{O}}{\partial \sigma_{sj}^2} = \sum_{i=1}^{N} \frac{\gamma_{ij}(s)}{\sigma_{sj}^2} \left(1 - \frac{3(x_{ij} - \mu_{sj})^2}{\sigma_{sj}^2} \right), \text{ and}$$

$$\frac{\partial^2 \mathcal{O}}{\partial \lambda_{sj}^2} = -\sum_{i=1}^{N} \gamma_{ij}(s) \frac{x_{ij}}{\lambda_{sj}^2}.$$

Appendix B. Supplementary Tables

Table B1

Average Bias of the Probability-Based Parameter Estimates ($|\mathcal{S}| = 3$)

				π_0			$\pi_{ss'}$			ϕ			
Init	Tr	Δ	N	RS1	RS2	RS3	RS1	RS2	RS3	RC1	RC2	RC3	RC4
Bl	St	Mod	100	-.004	.000	.004	-.001	.000	.001	.000	.001	.000	-.001
			300	.002	-.002	.000	.000	.000	.000	.000	.000	.000	-.001
			500	.002	-.003	.000	.000	.000	.000	.000	.000	.000	-.001
		Lrg	100	.000	.000	.000	.000	.000	.000	.000	.000	.000	-.001
			300	.001	-.002	.001	.000	.000	.000	.000	.000	.000	.000
			500	.002	-.002	.000	.000	.000	.000	.000	.000	.000	.000
	Unst	Mod	100	-.018	.011	.006	-.008	.003	.005	-.001	.001	.001	-.001
			300	-.009	.006	.003	-.005	.002	.003	.000	.001	.001	-.001
			500	-.012	.009	.003	-.005	.002	.003	-.001	.001	.001	-.001
		Lrg	100	-.002	.002	.001	-.001	.002	.000	.000	.000	.000	.000
			300	-.002	.000	.002	-.001	.000	.001	.000	.000	.000	.000
			500	.000	-.001	.001	-.001	.000	.000	.000	.000	.000	.000
Sk	St	Mod	100	.052	-.031	-.021	-.006	.002	.004	.001	-.001	.000	.001
			300	.025	-.012	-.013	-.002	.001	.001	.000	.000	.000	.000
			500	.020	-.009	-.011	-.002	.000	.001	.000	.000	.000	.000
		Lrg	100	.007	-.004	-.003	.000	.000	.001	.000	.000	.000	.000
			300	.007	-.003	-.004	.000	.000	.000	.000	.000	.000	.000
			500	.005	-.002	-.003	.000	.000	.000	.000	.000	.000	.000
	Unst	Mod	100	.102	-.044	-.057	-.010	.004	.006	.000	.000	.000	.000
			300	.090	-.043	-.047	-.006	.003	.003	.000	.000	.000	.000
			500	.087	-.042	-.045	-.005	.003	.003	.000	.000	.000	.000
		Lrg	100	.028	-.012	-.016	.000	.000	.000	.000	.000	.001	.000
			300	.020	-.007	-.013	.000	-.001	.000	.000	.000	.000	.000
			500	.017	-.007	-.010	.000	.000	.000	.000	.000	.000	.000

Note. π_0: Initial state probabilities. $\pi_{ss'}$: State transition probabilities. ϕ: Response probabilities for ordinal outcomes. Init: Initial state distribution (Bl: Balanced, Sk: Skewed). Tr: State transition scenarios (St: Stable (stayer probability = .9), Unst: Unstable (.7)). Δ: Difference in the emission parameters (Mod: Moderate (e.g., $\Delta\mu = .5$), Lrg: Large (1.0)). N: Sample size. RS: Reference state. RC: Reference score category. Biases were averaged over the remaining categories.

Table B2

Average Bias of the Model Parameter Estimates ($|\mathcal{S}| = 5$)

			Balanced Initial State Distribution						Skewed Initial State Distribution					
Tr	Δ	N	π_0	$\pi_{ss'}$	ϕ	μ	σ	λ	π_0	$\pi_{ss'}$	ϕ	μ	σ	λ
St	Mod	300	-.001	.000	-.001	.002	-.007	.004	.027	-.002	.000	.005	-.005	.002
		500	-.001	.000	-.002	.003	-.004	.001	.026	-.002	.000	.006	-.002	.009
		1000	-.001	.000	-.001	.002	-.002	.001	.024	-.002	.000	.004	.001	.004
	Lrg	300	.002	-.001	-.002	.009	.005	.000	.021	-.007	-.001	.051	.012	.073
		500	.001	-.001	-.002	.006	.006	.000	.020	-.008	.000	.062	.015	.102
		1000	.001	-.001	-.002	.005	.009	-.003	.016	-.007	.000	.061	.015	.105
Unst	Mod	300	.001	.000	-.001	.006	-.008	.014	.067	-.001	.000	.010	-.008	.014
		500	.002	.001	-.001	.007	-.003	.013	.071	-.001	.000	.009	-.002	.012
		1000	.002	.001	-.001	.008	.001	.015	.073	.000	.000	.008	.002	.015
	Lrg	300	.005	.000	-.001	.010	.006	.003	.029	.000	-.001	.016	.008	.017
		500	.005	.000	-.001	.008	.008	.002	.031	-.001	.000	.017	.011	.014
		1000	.005	.000	-.001	.008	.010	.005	.030	-.001	.000	.014	.013	.011

Note. Tr: State transition scenarios (St: Stable (stayer probability = .9), Unst: Unstable (.7)). Δ: Difference in the emission parameters (Mod: Moderate (e.g., $\Delta\,\mu = .5$), Lrg: Large (1.0)). N: Sample size. π_0: Initial state probabilities. $\pi_{ss'}$: State transition probabilities. ϕ: Response probabilities for ordinal outcomes. μ: Location parameter for continuous outcomes. σ: Scale parameter for continuous outcomes. λ: Rate parameter for count outcomes. The average biases for π_0, $\pi_{ss\prime}$, and ϕ were obtained by treating the first category as a baseline and averaging over the remaining categories.

Table B3

Average Bias of the Probability-Based Parameter Estimates ($|\mathcal{S}| = 5$)

				π_0					$\pi_{ss'}$					ϕ			
Init	Tr	Δ	N	RS1	RS2	RS3	RS4	RS5	RS1	RS2	RS3	RS4	RS5	RC1	RC2	RC3	RC4
Bl	St	Mod	300	-.001	.000	.000	-.001	.001	.000	.000	.001	.000	-.001	-.001	.000	.001	.001
			500	-.001	-.001	.002	.000	.000	.000	.000	.001	.000	-.001	-.002	-.001	.001	.002
			1000	-.001	.000	.001	.000	.001	.000	.000	.001	.001	-.001	-.001	-.001	.001	.002
		Lrg	300	.002	-.001	.005	-.001	-.005	-.001	-.001	.004	.001	-.004	-.002	-.001	.001	.002
			500	.001	-.001	.006	.000	-.006	-.001	.000	.004	.001	-.004	-.002	-.001	.001	.002
			1000	.001	-.002	.007	.001	-.008	-.001	-.001	.005	.001	-.005	-.002	-.001	.001	.002
	Unst	Mod	300	.001	-.003	.003	.001	-.002	.000	.000	.000	-.001	.000	-.001	.000	.001	.000
			500	.002	-.003	.002	-.001	-.001	.001	.001	.000	-.002	.001	-.001	.000	.001	.001
			1000	.002	-.003	.002	.000	-.001	.001	.000	.000	-.001	.000	-.001	.000	.001	.001
		Lrg	300	.005	-.002	.005	.000	-.008	.000	-.001	.003	.001	-.003	-.001	-.001	.000	.002
			500	.005	-.001	.003	-.002	-.006	.000	.000	.002	.000	-.002	-.001	-.001	.000	.002
			1000	.005	.000	.004	-.003	-.005	.000	.000	.002	.000	-.002	-.001	-.001	.000	.002
Sk	St	Mod	300	.027	-.006	-.005	-.010	-.005	-.002	.000	.002	.001	-.001	.000	-.001	-.001	.001
			500	.026	-.007	-.004	-.009	-.006	-.002	.000	.001	.001	.000	.000	-.001	.000	.002
			1000	.024	-.006	-.005	-.007	-.006	-.002	.000	.001	.001	.000	.000	-.001	.000	.002
		Lrg	300	.021	-.006	-.002	-.010	-.003	-.007	.007	.002	-.001	-.001	-.001	-.003	-.001	.004
			500	.020	-.007	.001	-.010	-.004	-.008	.006	.002	.002	-.002	.000	-.003	-.001	.004
			1000	.016	-.005	.002	-.009	-.005	-.007	.007	.004	.001	-.004	.000	-.003	-.001	.004
	Unst	Mod	300	.067	-.018	-.015	-.018	-.016	-.001	.000	.001	-.001	.000	.000	-.001	.000	.001
			500	.071	-.019	-.017	-.018	-.017	-.001	.000	.001	-.001	.001	.000	-.001	.000	.001
			1000	.073	-.020	-.017	-.019	-.017	.000	.000	.000	-.001	.001	.000	-.001	.000	.001
		Lrg	300	.029	-.010	-.005	-.007	-.007	.000	-.001	.002	.001	-.002	-.001	-.002	.000	.002
			500	.031	-.010	-.006	-.008	-.007	-.001	-.001	.003	.002	-.003	.000	-.002	-.001	.002
			1000	.030	-.009	-.004	-.008	-.008	-.001	-.001	.004	.001	-.004	.000	-.002	-.001	.002

Note. π_0: Initial state probabilities. $\pi_{ss'}$: State transition probabilities. ϕ: Response probabilities for ordinal outcomes. Init: Initial state distribution (Bl: Balanced, Sk: Skewed). Tr: State transition scenarios (St: Stable (stayer probability = .9), Unst: Unstable (.7)). Δ: Difference in the emission parameters (Mod: Moderate (e.g., $\Delta\,\mu$ = .5), Lrg: Large (1.0)). N: Sample size. RS: Reference state. RC: Reference score category. Biases were averaged over the remaining categories.

Table B4

Root Mean Squared Error of the Model Parameter Estimates ($|\mathcal{S}| = 5$)

			Balanced Initial State Distribution						Skewed Initial State Distribution					
Tr	Δ	N	π_0	$\pi_{ss'}$	ϕ	μ	σ	λ	π_0	$\pi_{ss'}$	ϕ	μ	σ	λ
St	Mod	300	.049	.290	.146	.232	.053	.397	.071	.254	.151	.240	.062	.446
		500	.045	.287	.138	.233	.046	.348	.066	.252	.140	.231	.052	.375
		1000	.045	.285	.131	.228	.037	.299	.062	.245	.130	.228	.043	.321
	Lrg	300	.069	.233	.145	.236	.061	.432	.070	.202	.154	.334	.084	.672
		500	.064	.222	.135	.235	.052	.372	.070	.207	.145	.343	.078	.619
		1000	.062	.215	.126	.230	.045	.319	.068	.187	.133	.327	.064	.538
Unst	Mod	300	.055	.215	.150	.262	.068	.470	.153	.217	.153	.266	.072	.494
		500	.052	.214	.138	.258	.058	.400	.161	.216	.141	.263	.061	.426
		1000	.048	.215	.130	.253	.050	.338	.163	.216	.131	.258	.053	.361
	Lrg	300	.066	.179	.140	.235	.065	.434	.072	.176	.143	.263	.072	.480
		500	.063	.174	.129	.228	.055	.365	.074	.176	.134	.259	.066	.424
		1000	.062	.174	.121	.223	.048	.314	.071	.175	.125	.254	.058	.374

Note. Tr: State transition scenarios (St: Stable (stayer probability = .9), Unst: Unstable (.7)). Δ: Difference in the emission parameters (Mod: Moderate (e.g., $\Delta\,\mu = .5$), Lrg: Large (1.0)). N: Sample size. π_0: Initial state probabilities. $\pi_{ss'}$: State transition probabilities. ϕ: Response probabilities for ordinal outcomes. μ: Location parameter for continuous outcomes. σ: Scale parameter for continuous outcomes. λ: Rate parameter for count outcomes.

Table B5

Average Standard Errors of the Model Parameter Estimates

			Balanced Initial State Distribution						Skewed Initial State Distribution					
Tr	Δ	N	π_0	$\pi_{ss'}$	ϕ	μ	σ	λ	π_0	$\pi_{ss'}$	ϕ	μ	σ	λ
								$\lvert\mathcal{S}\rvert = 3$						
St	Mod	100	.064	.021	.088	.051	.036	.298	.037	.021	.129	.052	.037	.308
		300	.034	.012	.050	.030	.021	.172	.016	.012	.051	.031	.022	.177
		500	.026	.009	.039	.023	.016	.133	.013	.009	.039	.024	.017	.137
	Lrg	100	.058	.020	.091	.052	.037	.288	.027	.020	.162	.055	.039	.299
		300	.033	.012	.051	.030	.021	.166	.016	.012	.278	.031	.022	.171
		500	.026	.009	.039	.023	.017	.128	.012	.009	.040	.024	.017	.132
Unst	Mod	100	.055	.023	.086	.048	.034	.299	.049	.023	.126	.048	.034	.300
		300	.032	.013	.050	.028	.020	.172	.028	.013	.050	.028	.020	.173
		500	.025	.010	.039	.022	.016	.133	.021	.010	.039	.022	.016	.134
	Lrg	100	.059	.022	.088	.052	.037	.287	.034	.023	.212	.053	.037	.293
		300	.034	.013	.050	.030	.022	.166	.018	.013	.093	.031	.022	.168
		500	.026	.010	.039	.024	.017	.128	.014	.010	.047	.024	.017	.130
								$\lvert\mathcal{S}\rvert = 5$						
St	Mod	300	.025	.012	.063	.038	.027	.223	.019	.012	.064	.039	.027	.224
		500	.020	.010	.050	.030	.021	.172	.015	.010	.050	.030	.021	.173
		1000	.014	.007	.035	.022	.015	.122	.010	.007	.035	.022	.015	.123
	Lrg	300	.031	.012	.064	.041	.029	.215	.019	.012	.065	.041	.030	.218
		500	.024	.009	.050	.032	.023	.166	.015	.009	.051	.033	.023	.169
		1000	.017	.007	.036	.023	.016	.117	.011	.007	.036	.023	.016	.120
Unst	Mod	300	.020	.013	.064	.038	.027	.223	.016	.013	.064	.038	.027	.225
		500	.016	.010	.050	.030	.021	.173	.012	.010	.050	.030	.021	.174
		1000	.011	.007	.036	.022	.015	.122	.009	.007	.036	.022	.015	.123
	Lrg	300	.027	.013	.064	.041	.029	.215	.017	.013	.064	.041	.030	.216
		500	.021	.010	.050	.032	.023	.166	.013	.010	.050	.032	.023	.167
		1000	.015	.007	.035	.023	.016	.118	.009	.007	.036	.023	.016	.118

Note. Tr: State transition scenarios (St: Stable (stayer probability = .9), Unst: Unstable (.7)). Δ: Difference in the emission parameters (Mod: Moderate (e.g., $\Delta\mu = .5$), Lrg: Large (1.0)). N: Sample size. π_0: Initial state probabilities. $\pi_{ss'}$: State transition probabilities. ϕ: Response probabilities for ordinal outcomes. μ: Location parameter for continuous outcomes. σ: Scale parameter for continuous outcomes. λ: Rate parameter for count outcomes.

Table B6

Average Absolute Bias and State Recovery Rate of the MI Model Fit to the MI Data

			Model Parameter Recovery												State Recovery	
			Balanced Initial State Distribution						Skewed Initial State Distribution							
Tr	Δ	N	π_0	$\pi_{ss'}$	ϕ	μ	σ	λ	π_0	$\pi_{ss'}$	ϕ	μ	σ	λ	BL	SK
St	Mod	100	.051	.060	.035	.050	.014	.181	.125	.059	.035	.053	.017	.179	.799	.786
		300	.032	.051	.029	.046	.012	.155	.122	.056	.030	.047	.014	.169	.804	.784
		500	.026	.051	.027	.044	.010	.144	.115	.052	.028	.046	.013	.160	.806	.790
	Lrg	100	.041	.010	.014	.010	.007	.057	.030	.010	.014	.010	.008	.053	.977	.982
		300	.022	.007	.008	.006	.004	.034	.017	.006	.008	.006	.004	.033	.979	.982
		500	.017	.006	.007	.005	.003	.027	.014	.005	.006	.005	.003	.024	.979	.982
Unst	Mod	100	.054	.083	.034	.078	.025	.202	.170	.083	.041	.091	.024	.236	.730	.690
		300	.031	.080	.030	.076	.023	.176	.169	.080	.037	.090	.022	.215	.730	.691
		500	.027	.080	.030	.075	.023	.178	.167	.081	.035	.090	.022	.209	.730	.691
	Lrg	100	.042	.016	.014	.013	.009	.058	.031	.016	.014	.011	.008	.056	.952	.950
		300	.023	.010	.008	.007	.006	.039	.018	.010	.008	.007	.005	.034	.953	.951
		500	.018	.009	.006	.006	.005	.030	.013	.008	.006	.006	.004	.028	.953	.951

Note. Tr: State transition scenarios (St: Stable (stayer probability = .9), Unst: Unstable (.7)). Δ: Difference in the emission parameters (Mod: Moderate (e.g., $\Delta\,\mu$ = .5), Lrg: Large (1.0)). N: Sample size. π_0: Initial state probabilities. $\pi_{ss'}$: State transition probabilities. ϕ: Response probabilities for ordinal outcomes. μ: Location parameter for continuous outcomes. σ: Scale parameter for continuous outcomes. λ: Rate parameter for count outcomes. BL: Balanced initial state distribution. SK: Skewed initial state distribution. The number of latent states was fixed at $|\mathcal{S}| = 3$.

Table B7

Average Absolute Bias and State Recovery Rate of the MNI Model Fit to the MNI Data

			Model Parameter Recovery												State Recovery	
			Balanced Initial State Distribution						Skewed Initial State Distribution							
Tr	Δ	N	π_0	$\pi_{ss'}$	ϕ	μ	σ	λ	π_0	$\pi_{ss'}$	ϕ	μ	σ	λ	BL	SK
St	Mod	100	.055	.039	.069	.058	.039	.284	.085	.041	.074	.065	.043	.312	.888	.882
		300	.030	.017	.038	.033	.021	.163	.041	.017	.040	.034	.022	.169	.919	.922
		500	.022	.017	.030	.027	.016	.128	.032	.014	.031	.026	.017	.130	.921	.926
	Lrg	100	.039	.009	.060	.044	.032	.234	.029	.009	.063	.049	.036	.254	.982	.981
		300	.022	.005	.034	.025	.018	.133	.020	.005	.036	.028	.020	.145	.984	.983
		500	.019	.004	.027	.019	.014	.103	.015	.004	.028	.021	.015	.112	.984	.983
Unst	Mod	100	.089	.083	.082	.088	.052	.364	.146	.082	.084	.090	.054	.373	.752	.749
		300	.062	.047	.048	.053	.028	.213	.125	.044	.049	.054	.030	.220	.800	.797
		500	.055	.042	.038	.044	.022	.170	.121	.038	.039	.045	.024	.177	.810	.806
	Lrg	100	.044	.015	.061	.048	.037	.251	.051	.015	.062	.051	.039	.257	.945	.946
		300	.023	.009	.035	.028	.021	.143	.033	.009	.036	.029	.022	.146	.951	.953
		500	.020	.007	.027	.022	.016	.111	.027	.007	.028	.023	.017	.112	.952	.954

Note. Tr: State transition scenarios (St: Stable (stayer probability = .9), Unst: Unstable (.7)). Δ: Difference in the emission parameters (Mod: Moderate (e.g., $\Delta\,\mu$ = .5), Lrg: Large (1.0)). N: Sample size. π_0: Initial state probabilities. $\pi_{ss'}$: State transition probabilities. ϕ: Response probabilities for ordinal outcomes. μ: Location parameter for continuous outcomes. σ: Scale parameter for continuous outcomes. λ: Rate parameter for count outcomes. BL: Balanced initial state distribution. SK: Skewed initial state distribution. The number of latent states was fixed at $|\mathcal{S}| = 3$.

Table B8

Average Bias of the MNI and MI Model Parameter Estimates When Ill-Fitted

			Balanced Initial State Distribution						Skewed Initial State Distribution					
Tr	Δ	N	π_0	$\pi_{ss'}$	ϕ	μ	σ	λ	π_0	$\pi_{ss'}$	ϕ	μ	σ	λ
			MNI Model Fit to the MI Data (Overfit)											
St	Mod	100	.005	.006	-.001	.000	-.019	-.002	-.007	.005	-.001	.004	-.018	-.014
		300	.009	.005	.000	.001	-.009	.001	-.010	.004	.000	.004	-.009	-.010
		500	.009	.005	.000	.000	-.007	.000	-.010	.005	.000	.004	-.007	-.011
	Lrg	100	.002	.001	-.001	.000	-.007	-.002	-.018	.001	-.001	-.004	-.007	.008
		300	.000	.000	.000	.000	-.003	-.002	-.022	.000	.000	-.004	-.004	.005
		500	.002	.000	.000	.000	-.002	.001	-.021	.000	.000	-.003	-.003	.007
Unst	Mod	100	.002	-.001	.000	.004	-.026	.005	-.008	-.002	.000	.002	-.026	-.004
		300	-.002	.000	.000	.002	-.019	-.001	-.011	-.001	.000	.000	-.019	-.009
		500	-.002	-.001	.000	.002	-.019	.002	-.011	-.001	.000	.001	-.018	-.004
	Lrg	100	.002	.000	-.001	.002	-.005	-.001	-.017	.000	-.001	.001	-.004	-.004
		300	.002	.001	.000	.001	.000	.003	-.018	.001	-.001	.000	.001	-.001
		500	.003	.001	.000	.002	.001	.002	-.017	.001	-.001	.000	.002	-.003
			MI Model Fit to the MNI Data (Underfit)											
St	Mod	100	.000	-.001	.000	-.002	.106	-.005	.062	-.006	.001	-.002	.097	-.002
		300	-.006	.000	.000	.002	.109	.004	.060	-.006	.000	-.003	.101	-.005
		500	-.005	.000	.000	.002	.109	.002	.063	-.006	.001	-.002	.101	-.003
	Lrg	100	.000	.000	.001	.001	.115	-.007	-.006	.000	.000	-.001	.112	-.003
		300	.004	.000	.000	.000	.116	-.001	-.003	.000	.000	.000	.113	-.001
		500	.004	.000	.000	.000	.116	.000	-.004	.000	.000	-.001	.113	-.002
Unst	Mod	100	-.037	-.016	.000	.005	.062	-.001	.080	-.011	.001	.004	.060	.007
		300	-.027	-.016	.000	.004	.061	.008	.085	-.013	.001	.000	.059	.002
		500	-.030	-.016	.000	.005	.061	.009	.083	-.012	.001	.000	.059	.003
	Lrg	100	-.003	-.002	-.001	-.001	.121	-.006	-.003	.001	.000	.006	.111	.010
		300	-.005	-.002	.000	.000	.120	.000	.002	.001	.000	.005	.112	.008
		500	-.005	-.001	.000	-.001	.121	-.002	.001	.001	.000	.005	.112	.009

Note. Tr: State transition scenarios (St: Stable (stayer probability = .9), Unst: Unstable (.7)). Δ: Difference in the emission parameters (Mod: Moderate (e.g., $\Delta\mu = .5$), Lrg: Large (1.0)). N: Sample size. π_0: Initial state probabilities. $\pi_{ss'}$: State transition probabilities. ϕ: Response probabilities for ordinal outcomes. μ: Location parameter for continuous outcomes. σ: Scale parameter for continuous outcomes. λ: Rate parameter for count outcomes. The number of latent states was fixed at $|\mathcal{S}| = 3$. The average biases for π_0, $\pi_{ss'}$, and ϕ were obtained by treating the first category as a baseline and averaging over the remaining categories.

Table B9

Root Mean Squared Error of the MNI and MI Model Parameter Estimates When Ill-Fitted

			Balanced Initial State Distribution						Skewed Initial State Distribution					
Tr	Δ	N	π_0	$\pi_{ss'}$	ϕ	μ	σ	λ	π_0	$\pi_{ss'}$	ϕ	μ	σ	λ
			MNI Model Fit to the MI Data (Overfit)											
St	Mod	100	.067	.109	.104	.104	.055	.476	.082	.110	.110	.131	.058	.487
		300	.036	.084	.069	.077	.032	.315	.059	.083	.077	.106	.034	.332
		500	.032	.080	.059	.070	.026	.270	.056	.078	.068	.100	.028	.289
	Lrg	100	.048	.015	.076	.056	.041	.317	.072	.015	.082	.097	.044	.337
		300	.026	.008	.044	.032	.023	.185	.054	.007	.051	.077	.026	.202
		500	.019	.007	.034	.025	.018	.143	.049	.006	.042	.070	.021	.161
Unst	Mod	100	.059	.107	.108	.127	.066	.496	.073	.110	.111	.148	.068	.514
		300	.037	.098	.070	.104	.043	.337	.052	.098	.075	.127	.044	.359
		500	.031	.097	.061	.099	.037	.297	.045	.097	.066	.122	.039	.321
	Lrg	100	.046	.022	.079	.062	.048	.331	.070	.021	.085	.100	.049	.360
		300	.025	.013	.045	.035	.027	.192	.050	.013	.052	.076	.029	.227
		500	.020	.011	.035	.027	.020	.147	.047	.011	.043	.069	.023	.186
			MI Model Fit to the MNI Data (Underfit)											
St	Mod	100	.147	.073	.087	.296	.114	.319	.182	.103	.088	.296	.106	.327
		300	.131	.065	.085	.295	.116	.309	.173	.085	.085	.295	.110	.315
		500	.127	.064	.085	.294	.116	.306	.178	.087	.085	.295	.109	.313
	Lrg	100	.054	.012	.085	.291	.122	.299	.045	.012	.084	.288	.119	.303
		300	.040	.008	.083	.290	.122	.293	.035	.008	.082	.288	.120	.297
		500	.039	.007	.083	.290	.122	.291	.032	.006	.082	.288	.120	.296
Unst	Mod	100	.179	.146	.089	.305	.074	.316	.199	.143	.090	.303	.075	.325
		300	.168	.152	.087	.304	.072	.306	.197	.148	.089	.302	.073	.316
		500	.166	.151	.087	.304	.072	.303	.193	.148	.088	.302	.073	.314
	Lrg	100	.081	.021	.082	.296	.127	.303	.051	.021	.084	.288	.118	.300
		300	.070	.015	.081	.296	.127	.297	.046	.015	.083	.288	.119	.295
		500	.069	.013	.081	.296	.127	.296	.045	.013	.083	.288	.119	.293

Note. Tr: State transition scenarios (St: Stable (stayer probability = .9), Unst: Unstable (.7)). Δ: Difference in the emission parameters (Mod: Moderate (e.g., $\Delta\mu = .5$), Lrg: Large (1.0)). N: Sample size. π_0: Initial state probabilities. $\pi_{ss'}$: State transition probabilities. ϕ: Response probabilities for ordinal outcomes. μ: Location parameter for continuous outcomes. σ: Scale parameter for continuous outcomes. λ: Rate parameter for count outcomes. The number of latent states was fixed at $|\mathcal{S}| = 3$.

Table B10

Average Standard Errors of the MNI and MI Model Parameter Estimates When Ill-Fitted

			Balanced Initial State Distribution						Skewed Initial State Distribution					
Tr	Δ	N	π_0	$\pi_{ss'}$	ϕ	μ	σ	λ	π_0	$\pi_{ss'}$	ϕ	μ	σ	λ
			MNI Model Fit to the MI Data (Overfit)											
St	Mod	100	.059	.020	.090	.050	.035	.300	.060	.020	.090	.050	.035	.300
		300	.035	.011	.051	.030	.021	.173	.034	.011	.051	.030	.021	.173
		500	.027	.009	.039	.023	.016	.134	.026	.009	.039	.023	.016	.134
	Lrg	100	.057	.019	.091	.051	.036	.288	.053	.019	.092	.052	.037	.291
		300	.033	.011	.051	.030	.021	.165	.031	.011	.051	.030	.021	.167
		500	.026	.008	.039	.023	.016	.128	.024	.008	.039	.023	.016	.129
Unst	Mod	100	.058	.022	.087	.048	.034	.301	.058	.022	.087	.048	.034	.300
		300	.033	.013	.050	.028	.020	.173	.032	.013	.050	.028	.020	.173
		500	.025	.010	.039	.022	.015	.134	.025	.010	.039	.022	.016	.134
	Lrg	100	.058	.021	.089	.052	.037	.290	.056	.021	.089	.052	.037	.290
		300	.033	.012	.050	.030	.022	.167	.032	.012	.050	.030	.022	.167
		500	.026	.010	.039	.023	.017	.129	.025	.010	.039	.024	.017	.129
			MI Model Fit to the MNI Data (Underfit)											
St	Mod	100	.070	.019	.020	.016	.011	.067	.040	.020	.020	.016	.011	.068
		300	.041	.011	.011	.009	.007	.039	.027	.012	.012	.009	.007	.039
		500	.031	.009	.009	.007	.005	.030	.020	.009	.009	.007	.005	.030
	Lrg	100	.058	.019	.020	.016	.011	.064	.022	.019	.020	.017	.012	.066
		300	.034	.011	.011	.009	.007	.037	.014	.011	.012	.010	.007	.038
		500	.026	.008	.009	.007	.005	.029	.010	.009	.009	.007	.005	.029
Unst	Mod	100	.069	.022	.020	.014	.010	.067	.049	.022	.020	.014	.010	.067
		300	.042	.013	.011	.008	.006	.039	.031	.013	.011	.008	.006	.039
		500	.032	.010	.009	.006	.004	.030	.025	.010	.009	.006	.004	.030
	Lrg	100	.062	.021	.020	.016	.012	.064	.025	.022	.020	.016	.011	.065
		300	.036	.012	.011	.009	.007	.037	.014	.012	.011	.009	.007	.037
		500	.028	.010	.009	.007	.005	.029	.011	.010	.009	.007	.005	.029

Note. Tr: State transition scenarios (St: Stable (stayer probability = .9), Unst: Unstable (.7)). Δ: Difference in the emission parameters (Mod: Moderate (e.g., $\Delta\,\mu = .5$), Lrg: Large (1.0)). N: Sample size. π_0: Initial state probabilities. $\pi_{ss'}$: State transition probabilities. ϕ: Response probabilities for ordinal outcomes. μ: Location parameter for continuous outcomes. σ: Scale parameter for continuous outcomes. λ: Rate parameter for count outcomes. The number of latent states was fixed at $|\mathcal{S}| = 3$.

Table B11

Emission Parameter Values from the S07 Booklet Data

			Response Score			Interaction Time		Number of Actions	
Item ID	Type	St	ϕ_{sj0}	ϕ_{sj1}	ϕ_{sj2}	μ_{sj}	σ_{sj}	μ_{sj}	σ_{sj}
S627Q01	SMC	1	.575 (.028)	.425 (.024)	-	3.841 (.014)	.371 (.010)	.521 (.018)	.473 (.012)
		2	.628 (.038)	.372 (.030)	-	3.971 (.037)	.759 (.026)	.930 (.036)	.740 (.025)
S627Q03	CMC	1	.096 (.014)	.904 (.042)	-	3.895 (.016)	.358 (.011)	1.421 (.004)	.082 (.003)
		2	.335 (.023)	.665 (.033)	-	4.024 (.022)	.549 (.015)	1.853 (.020)	.490 (.014)
S627Q04	CMC	1	.194 (.022)	.806 (.044)	-	3.711 (.017)	.342 (.012)	1.650 (.004)	.076 (.003)
		2	.352 (.022)	.648 (.030)	-	3.869 (.019)	.503 (.013)	1.876 (.010)	.268 (.007)
S635Q01	CMC	1	.185 (.023)	.498 (.038)	.317 (.030)	3.990 (.020)	.368 (.014)	1.701 (.006)	.116 (.004)
		2	.266 (.018)	.471 (.025)	.263 (.018)	4.301 (.018)	.503 (.013)	2.059 (.016)	.439 (.011)
S635Q02	CMC	1	.510 (.042)	.490 (.041)	-	3.669 (.031)	.532 (.022)	2.146 (.019)	.332 (.014)
		2	.227 (.016)	.773 (.030)	-	4.297 (.014)	.405 (.010)	2.566 (.017)	.480 (.012)
S635Q03	OR	1	.934 (.059)	.066 (.016)	-	4.236 (.042)	.679 (.030)	3.377 (.084)	1.366 (.059)
		2	.408 (.022)	.592 (.026)	-	4.894 (.013)	.397 (.010)	5.038 (.018)	.538 (.013)
S635Q04	OR	1	.626 (.044)	.293 (.030)	.081 (.027)	3.862 (.04)	.695 (.028)	2.333 (.033)	.571 (.023)
		2	.270 (.018)	.284 (.018)	.446 (.023)	4.481 (.014)	.391 (.010)	2.726 (.013)	.390 (.010)
S635Q05	OR	1	.986 (.054)	.013 (.004)	.001 (.002)	4.139 (.042)	.770 (.029)	2.746 (.065)	1.206 (.046)
		2	.656 (.029)	.142 (.013)	.202 (.016)	5.204 (.015)	.410 (.010)	5.208 (.021)	.597 (.015)
S603Q01	SMC	1	.656 (.045)	.344 (.033)	-	3.591 (.059)	1.058 (.042)	1.301 (.034)	.607 (.024)
		2	.102 (.011)	.898 (.033)	-	4.129 (.016)	.449 (.011)	.998 (.011)	.301 (.007)
S603Q02	OR	1	.930 (.058)	.070 (.016)	-	3.643 (.060)	.996 (.042)	2.646 (.093)	1.558 (.066)
		2	.528 (.025)	.472 (.023)	-	4.813 (.015)	.443 (.011)	4.958 (.021)	.627 (.015)
S603Q03	SMC	1	.666 (.048)	.334 (.033)	-	3.129 (.054)	.921 (.038)	1.283 (.034)	.583 (.024)
		2	.137 (.013)	.863 (.032)	-	3.451 (.014)	.396 (.010)	.997 (.010)	.276 (.007)
S603Q04	SMC	1	.731 (.051)	.269 (.030)	-	2.948 (.056)	.948 (.040)	1.180 (.031)	.519 (.021)
		2	.244 (.017)	.756 (.030)	-	3.445 (.014)	.396 (.010)	1.051 (.012)	.341 (.009)
S603Q05	SMC	1	.719 (.051)	.281 (.031)	-	2.930 (.053)	.884 (.037)	1.368 (.014)	.241 (.010)
		2	.279 (.018)	.721 (.029)	-	3.908 (.015)	.435 (.011)	1.453 (.014)	.416 (.010)
S602Q01	CMC	1	.524 (.046)	.476 (.044)	-	3.469 (.059)	.941 (.042)	1.497 (.013)	.203 (.009)
		2	.122 (.012)	.878 (.031)	-	4.157 (.015)	.460 (.011)	1.619 (.011)	.318 (.008)
S602Q02	CMC	1	.946 (.063)	.054 (.015)	-	2.766 (.052)	.800 (.037)	1.337 (.036)	.547 (.025)
		2	.641 (.027)	.359 (.020)	-	4.219 (.017)	.502 (.012)	1.539 (.018)	.544 (.013)
S602Q03	OR	1	.968 (.066)	.032 (.015)	-	3.451 (.077)	1.153 (.055)	2.576 (.125)	1.864 (.088)
		2	.463 (.022)	.537 (.024)	-	4.564 (.016)	.477 (.011)	4.958 (.019)	.583 (.014)
S602Q04	CMC	1	.775 (.056)	.225 (.031)	-	3.289 (.064)	1.004 (.045)	1.926 (.028)	.436 (.019)
		2	.212 (.015)	.788 (.030)	-	3.981 (.014)	.414 (.010)	1.952 (.007)	.200 (.005)

Note. Item type: SMC (Simple multiple-choice), CMC (Complex multiple-choice), OR (Open response). St: State. ϕ_{sjm}: Probability of scoring m on item j at state s. μ_{sj}: Mean of the continuous outcome of item j at state s. σ_{sj}: Standard deviation of the continuous outcome of item j at state s. Within the parentheses are standard errors of the parameter estimates.

Table B12

Emission Parameter Values from the S09 Booklet Data

			Response Score			Interaction Time		Number of Actions	
Item ID	Type	St	ϕ_{sj0}	ϕ_{sj1}	ϕ_{sj2}	μ_{sj}	σ_{sj}	μ_{sj}	σ_{sj}
S649Q01	SMC	1	.701 (.076)	.299 (.049)	-	3.300 (.099)	1.096 (.070)	.618 (.047)	.523 (.033)
		2	.780 (.035)	.220 (.019)	-	4.060 (.020)	.504 (.014)	.329 (.015)	.384 (.011)
		3	.647 (.040)	.353 (.029)	-	4.366 (.021)	.431 (.015)	1.052 (.032)	.640 (.022)
S649Q02	OR	1	.963 (.085)	.037 (.017)	-	3.742 (.086)	.992 (.061)	2.991 (.142)	1.635 (.100)
		2	.944 (.038)	.056 (.009)	-	4.423 (.015)	.375 (.010)	4.531 (.021)	.530 (.015)
		3	.948 (.051)	.052 (.012)	-	5.113 (.020)	.379 (.014)	5.370 (.026)	.494 (.018)
S649Q03	CMC	1	.905 (.077)	.095 (.025)	-	3.125 (.033)	.409 (.024)	2.035 (.010)	.124 (.007)
		2	.762 (.034)	.238 (.019)	-	3.793 (.014)	.357 (.010)	2.138 (.008)	.195 (.005)
		3	.789 (.047)	.211 (.024)	-	4.257 (.026)	.486 (.018)	2.463 (.018)	.348 (.013)
S649Q04	SMC	1	.612 (.060)	.388 (.048)	-	3.015 (.056)	.728 (.040)	1.358 (.021)	.268 (.015)
		2	.545 (.029)	.455 (.027)	-	3.515 (.014)	.360 (.010)	1.256 (.006)	.165 (.005)
		3	.662 (.044)	.338 (.031)	-	3.999 (.027)	.507 (.019)	1.670 (.024)	.442 (.017)
S634Q01	CMC	1	.985 (.072)	.015 (.009)	-	3.964 (.039)	.541 (.028)	2.092 (.038)	.524 (.027)
		2	.773 (.036)	.227 (.019)	-	4.472 (.011)	.262 (.008)	2.043 (.013)	.324 (.009)
		3	.676 (.043)	.324 (.030)	-	4.938 (.014)	.274 (.010)	2.596 (.023)	.437 (.016)
S634Q02	CMC	1	.901 (.068)	.099 (.022)	.000 (.004)	3.738 (.038)	.534 (.027)	2.100 (.027)	.382 (.019)
		2	.610 (.032)	.327 (.024)	.064 (.010)	4.435 (.014)	.330 (.010)	2.427 (.011)	.264 (.008)
		3	.262 (.026)	.437 (.034)	.300 (.028)	5.022 (.016)	.310 (.011)	2.950 (.017)	.326 (.012)
S634Q03	OR	1	.995 (.068)	.005 (.005)	-	3.884 (.048)	.706 (.034)	1.926 (.067)	.984 (.048)
		2	.799 (.037)	.201 (.019)	-	4.699 (.013)	.309 (.009)	4.489 (.018)	.444 (.013)
		3	.629 (.042)	.371 (.032)	-	5.243 (.018)	.337 (.012)	5.335 (.022)	.427 (.016)
S634Q05	OR	1	.995 (.069)	.005 (.003)	-	3.820 (.058)	.844 (.041)	2.384 (.089)	1.287 (.063)
		2	.838 (.037)	.162 (.016)	-	4.766 (.015)	.377 (.011)	4.606 (.019)	.460 (.013)
		3	.570 (.040)	.430 (.035)	-	5.350 (.017)	.313 (.012)	5.438 (.022)	.407 (.015)
S634Q04	CMC	1	.826 (.055)	.174 (.043)	-	2.914 (.049)	.759 (.035)	1.252 (.046)	.711 (.032)
		2	.287 (.022)	.713 (.035)	-	3.261 (.012)	.285 (.008)	1.422 (.014)	.336 (.010)
		3	.541 (.041)	.459 (.038)	-	3.663 (.024)	.437 (.017)	1.711 (.027)	.483 (.019)
S620Q01	SMC	1	.434 (.045)	.566 (.051)	-	3.304 (.046)	.675 (.032)	.635 (.037)	.550 (.026)
		2	.042 (.008)	.958 (.039)	-	3.426 (.013)	.323 (.009)	.339 (.015)	.379 (.011)
		3	.099 (.018)	.901 (.053)	-	3.756 (.026)	.469 (.019)	.870 (.033)	.592 (.023)
S620Q02	CMC	1	.951 (.068)	.049 (.015)	-	3.226 (.065)	.934 (.046)	1.242 (.035)	.499 (.024)
		2	.664 (.033)	.336 (.023)	-	4.129 (.012)	.293 (.008)	1.319 (.017)	.414 (.012)
		3	.528 (.040)	.472 (.038)	-	4.492 (.017)	.308 (.012)	1.643 (.031)	.555 (.022)
S620Q04	OR	1	.996 (.074)	.004 (.005)	-	3.695 (.066)	.886 (.046)	2.548 (.115)	1.547 (.081)
		2	.641 (.031)	.359 (.023)	-	4.514 (.013)	.339 (.009)	4.530 (.019)	.484 (.013)
		3	.440 (.037)	.560 (.042)	-	5.166 (.018)	.326 (.013)	5.458 (.027)	.482 (.019)
S638Q01	CMC	1	.928 (.069)	.072 (.023)	-	3.117 (.073)	1.006 (.052)	1.325 (.034)	.472 (.024)
		2	.350 (.022)	.650 (.031)	-	3.954 (.014)	.373 (.010)	1.295 (.008)	.223 (.006)
		3	.534 (.044)	.466 (.041)	-	4.356 (.025)	.422 (.018)	1.873 (.041)	.676 (.029)
S638Q02	CMC	1	.694 (.063)	.306 (.042)	-	3.373 (.052)	.682 (.037)	1.615 (.019)	.249 (.014)
		2	.051 (.009)	.949 (.036)	-	3.543 (.013)	.347 (.009)	1.470 (.010)	.273 (.019)
		3	.329 (.036)	.671 (.054)	-	3.760 (.048)	.742 (.033)	1.818 (.034)	.516 (.017)
S638Q04	CMC	1	.883 (.072)	.117 (.026)	-	3.244 (.055)	.720 (.039)	1.848 (.020)	.259 (.014)
		2	.551 (.027)	.449 (.024)	-	3.612 (.012)	.318 (.008)	1.777 (.007)	.182 (.005)
		3	.726 (.056)	.274 (.034)	-	3.764 (.051)	.775 (.036)	2.003 (.044)	.674 (.031)
S638Q05	OR	1	.946 (.073)	.054 (.155)	-	3.191 (.063)	.826 (.045)	.926 (.023)	.300 (.016)
		2	.597 (.030)	.403 (.025)	-	3.890 (.019)	.492 (.014)	3.265 (.034)	.883 (.024)
		3	.635 (.044)	.365 (.033)	-	4.625 (.023)	.420 (.016)	4.881 (.028)	.512 (.020)

Note. Item type: SMC (Simple multiple-choice), CMC (Complex multiple-choice), OR (Open response). St: State. ϕ_{sjm}: Probability of scoring m on item j at state s. μ_{sj}: Mean of the continuous outcome of item j at state s. σ_{sj}: Standard deviation of the continuous outcome of item j at state s. Within the parentheses are standard errors of the parameter estimates.

Appendix C. Performance of the Model Fit Measures

The performance of the model fit measures was evaluated through a small Monte Carlo simulation study. To ensure adequate relevance to real data, we chose an example setting from one of the real assessments and examined the behavior of the fit measures in suggesting the models and latent dimensionality. We in particular chose PISA 2015 S09 booklet as a benchmark for replicating data given its more consistent patterns in the model and state choice (Table 9). The results from the analysis suggested that the measurement-noninvariant (MNI) model achieves the best fit when three latent states are assumed and the measurement-invariant (MI) model achieves the greatest fit under the five-state solution. The parameter values estimated from these conditions were used as generating parameters to yield two sets of 100 replication data, each under the MNI and MI configurations.

To evaluate the performance of the fit measures in identifying the underlying model, we fit two competing models—the MNI and MI models—to each simulated data and count the instances in which the fit measures correctly point to the generating model, that is, when the log-likelihood yields a higher value and the information criteria yield smaller values for the generating model. Table C1 reports percentages of replications that the fit measures correctly identified the generating model. When data were generated from the MNI model (with three latent states), all model fit measures correctly identified the MNI as a generating model. When data were generated from the MI model (with five latent states),

Table C1

Percent of Replications That Model Fit Measures Point to the Generating Model

Model	logLike	AIC	CAIC	BIC	ABIC
MNI	100%	100%	100%	100%	100%
MI	9%	86%	100%	100%	100%

Note. logLike: log-likelihood. AIC: Akaike information criterion. CAIC: Corrected AIC. BIC: Bayesian information criterion. ABIC: Adjusted BIC. MNI data were generated assuming three latent states, and MI data assuming five latent states as per Table 9.

CAIC, BIC, and ABIC properly identified the generating MI model while AIC led to a number of mis-identifications. The log-likelihood was found to perform distinctly subpar.

For evaluating the performance in identifying the underlying dimensionality, we again used the replication data from the MNI and MI models and fit latent Markov models with different numbers of latent states. Table C2 reports percentages of replications in which the fit measures correctly point to the generating number of latent states under each MNI and MI configuration. When data were generated from the MNI model with three latent states, the fit measures correctly identified the underlying dimensionality at around 80% rate. When data were generated from the MI model with five latent states, the underlying dimensionality was identified perfectly by all fit measures.

Table C2

Percent of Replications That Model Fit Measures Point to the Generating Number of Latent States

	MNI ($\|\mathcal{S}\| = 3$)					MI ($\|\mathcal{S}\| = 5$)				
$\|\mathcal{S}\|$	logLike	AIC	CAIC	BIC	ABIC	logLike	AIC	CAIC	BIC	ABIC
1	0%	0%	0%	0%	0%	0%	0%	0%	0%	0%
2	19%	19%	19%	20%	19%	0%	0%	0%	0%	0%
3	81%	81%	81%	80%	81%	0%	0%	0%	0%	0%
4	0%	0%	0%	0%	0%	0%	0%	0%	0%	0%
5	0%	0%	0%	0%	0%	100%	100%	100%	100%	100%

Note. $|\mathcal{S}|$: Number of latent states. logLike: log-likelihood. AIC: Akaike information criterion. CAIC: Corrected AIC. BIC: Bayesian information criterion. ABIC: Adjusted BIC. MNI data were generated assuming three latent states, and MI data assuming five latent states as per Table 9.

The observations from the experiment suggest that the fit measures considered in this study overall perform adequately well in identifying the underlying model and latent dimensionality while the AIC and log-likelihood may be used with caution in choosing the models.
